# Supplementary material for: dCas9 regulator to neutralize competition in CRISPRi circuits
Source: Nat Commun. 2021 Mar 16;12:1692. doi: 10.1038/s41467-021-21772-6 (PMC7966764; doi:10.1038/s41467-021-21772-6)
Supplement: Supplementary file 1 — Supplementary Information [file 41467_2021_21772_MOESM1_ESM.pdf]

dCas9 regulator to neutralize competition in CRISPRi circuits

Supplementary Information

Hsin-Ho Huang[1†], Massimo Bellato[2†], Yili Qian [1], Pablo Cárdenas [3], Lorenzo Pasotti [2], Paolo Magni [2], Domitilla Del Vecchio [1,4*]

[1] Department of Mechanical Engineering, Massachusetts Institute of Technology, Cambridge, MA, USA.
[2] Laboratory of Bioinformatics, Mathematical Modelling and Synthetic Biology, Department of Electrical, Computer and Biomedical Engineering, University of Pavia, Pavia, Italy
[3] Department of Biological Engineering, Massachusetts Institute of Technology, Cambridge, MA, USA.
[4] Synthetic Biology Center, Massachusetts Institute of Technology, Cambridge, MA, USA.

[†] These authors contributed equally to this work.
[*] Corresponding author.

Supplementary Note 1

Modeling framework

In Supplementary Note 1.1, we first establish a general modeling framework for CRISPRi-based genetic circuits. The specific models for the NOT gate and the cascade are described in Supplementary Note 1.2. In Supplementary Note 1.3, we provide mathematical analysis to guide the regulated dCas9 generator design and parameter tuning. These analysis results are used to educate experimental choices.

Supplementary Note 1.1 A general modeling framework for CRISPRi-based genetic circuits

In this section, we first describe the dynamics of the sgRNAs and the complexes they form, and then establish models for the dynamics of dCas9 protein and other proteins in the circuit.

sgRNA dynamics

We consider a CRISPRi-based genetic circuit composed of a set of sgRNAs g_i, where i takes value in an index set $\mathcal{I}$. Each sgRNA g_i can bind with apo-dCas9 (D) to form a dCas9-sgRNA complex c_i. These sgRNAs further fall into two complementary subsets $\mathcal{I}_1$ and $\mathcal{I}_2 = \mathcal{I} \setminus \mathcal{I}_1$. For sgRNA g_i such that $i \in \mathcal{I}_1$, the complex c_i can bind with its targeting site on promoter p_{t_i} to form a complex c_{ii}. Alternatively, for sgRNA g_i such that $i \in \mathcal{I}_2$, the complex c_i does not have a targeting site. These biomolecular processes can be described by the following chemical reactions:

$$\mathrm{g_i} + \mathrm{D} \underset{d_i}{\overset{a_i}{\rightleftharpoons}} \mathrm{c_i},\ \forall i \in \mathcal{I}, \qquad \mathrm{c_i} + \mathrm{p_{t_i}} \underset{k_{\mathrm{off}}^i}{\overset{k_{\mathrm{on}}^i}{\rightleftharpoons}} \mathrm{c_{ii}},\ \forall i \in \mathcal{I}_1. \tag{1}$$

dCas9 protein can dissociate from sgRNA even when the dCas9-sgRNA complex is bound to DNA. To model this phenomenon, for $i \in \mathcal{I}_1$, we also consider the reaction

$$\mathrm{c_{ii}} \xrightarrow{d_i} \mathrm{p_{t_i}} + \mathrm{D} + \mathrm{g_i}. \tag{2}$$

Let p_i represent the promoter transcribing sgRNA g_i, the production and decay of sgRNAs are described as:

$$\mathrm{p_i} \xrightarrow{u_i} \mathrm{p_i} + \mathrm{g_i}, \qquad \mathrm{g_i} \xrightarrow{\theta_i} \varnothing, \tag{3}$$

where u_i is the synthesis rate constant of sgRNA from a single promoter and θ_i is the degradation rate constant of sgRNA g_i. The magnitude of the synthesis rate constant u_i increases with the strength of the promoter. Additionally, we take into account the fact that all species are diluted at rate constant δ due to cell growth:

$$\mathrm{g_i}, \mathrm{c_i}, \mathrm{c_{ii}} \xrightarrow{\delta} \varnothing. \tag{4}$$

By mass action kinetics [S1], the chemical reactions in (1)-(4) can be modeled by the following ODEs:

$$\frac{\mathrm{d}}{\mathrm{d}t} g_i = u_i p_i - (\delta + \theta_i) g_i - a_i D g_i + d_i c_i + d_i c_{ii},\ \forall i \in \mathcal{I}_1, \tag{5a}$$

$$\frac{\mathrm{d}}{\mathrm{d}t} g_i = u_i p_i - (\delta + \theta_i) g_i - a_i D g_i + d_i c_i,\ \forall i \in \mathcal{I}_2, \tag{5b}$$

$$\frac{\mathrm{d}}{\mathrm{d}t} c_i = a_i D g_i - d_i c_i - \delta c_i - k_{\mathrm{on}}^i c_i p_{t_i} + k_{\mathrm{off}}^i c_{ii},\ \forall i \in \mathcal{I}_1, \tag{5c}$$

$$\frac{\mathrm{d}}{\mathrm{d}t} c_i = a_i D g_i - d_i c_i - \delta c_i,\ \forall i \in \mathcal{I}_2, \tag{5d}$$

$$\frac{\mathrm{d}}{\mathrm{d}t} c_{ii} = k_{\mathrm{on}}^i c_i p_{t_i} - k_{\mathrm{off}}^i c_{ii} - \delta c_{ii} - d_i c_{ii},\ \forall i \in \mathcal{I}_1. \tag{5e}$$

For $i \in \mathcal{I}_1$, let $\bar{c}_i := c_i + c_{ii}$ be the total amount of dCas9-sgRNA complex, using (5c) and (5e), we have

$$\frac{\mathrm{d}}{\mathrm{d}t}\bar{c}_i = a_i D g_i - d_i \bar{c}_i - \delta \bar{c}_i. \tag{6}$$

To obtain a reduced order model of (5) that facilitates analysis and educates design, we assume that binding and unbinding dynamics of the complexes c_i and c_{ii} are sufficiently fast compared to RNA and protein dynamics, and hence we assume their concentrations reach quasi-steady state (QSS). By setting the temporal derivatives in (5c)-(5e) to zero we obtain the QSS complex concentrations:

$$\bar{c}_i = \frac{D g_i}{K_i}, \qquad c_{ii} = \frac{c_i p_{t_i}}{Q_i},\ \forall i \in \mathcal{I}_1 \qquad c_i = \frac{D g_i}{K_i},\ \forall i \in \mathcal{I}_2, \tag{7}$$

where

$$K_i := \frac{d_i + \delta}{a_i} \qquad \text{and} \qquad Q_i := \frac{k_{\text{off}}^i + d_i + \delta}{k_{\text{on}}^i} \tag{8}$$

are the dissociation constants describing the binding between sgRNAs and apo-dCas9 protein, and between dCas9-sgRNA complex and the targeting promoter, respectively. Substituting (7) into (5a) and (5b), the dynamics of g_i can be re-written as:

$$\begin{aligned}\frac{\mathrm{d}}{\mathrm{d}t}g_i &= u_i p_i - (\delta + \theta_i) g_i - \delta \bar{c}_i,\ \forall i \in \mathcal{I}_1,\\ \frac{\mathrm{d}}{\mathrm{d}t}g_i &= u_i p_i - (\delta + \theta_i) g_i - \delta c_i,\ \forall i \in \mathcal{I}_2.\end{aligned} \tag{9}$$

The pool of total dCas9 protein is shared by all sgRNAs in the circuit. Let D_T represent the total dCas9 concentration, then dCas9 concentration follow the conservation law:

$$D_T = D + \sum_{i\in\mathcal{I}_1} \bar{c}_i + \sum_{i\in\mathcal{I}_2} c_i = D\left(1 + \sum_{i\in\mathcal{I}} \frac{g_i}{K_i}\right) \qquad \Rightarrow \qquad D = \frac{D_T}{1 + \sum_{i\in\mathcal{I}}(g_i/K_i)}. \tag{10}$$

Substituting (10) into (7), the QSS concentrations of the complexes in (7) can be re-written as:

$$\bar{c}_i = \frac{D_T \cdot (g_i/K_i)}{1 + \sum_{j\in\mathcal{I}}(g_j/K_j)},\ \forall i \in \mathcal{I}_1, \qquad c_i = \frac{D_T \cdot (g_i/K_i)}{1 + \sum_{j\in\mathcal{I}}(g_j/K_j)},\ \forall i \in \mathcal{I}_2. \tag{11}$$

For $i \in \mathcal{I}_1$, to find the extent of repression of g_i on its target promoter p_{t_i}, we need to compute the concentration of c_{ii}. To this end, suppose that $t_i = j$ for some $j \in \mathcal{I}$, we note that the concentration of p_j promoter follows the conservation law:

$$p_j^t = p_j + c_{ii}, \tag{12}$$

where p_j^t is the total concentration of the promoter driving the transcription of g_j. Substituting the QSS of c_{ii} in (7) into (12), we have

$$p_j\left(1 + \frac{c_i}{Q_i}\right) = p_j^t, \qquad \Rightarrow \qquad p_j(c_i) = \frac{Q_i p_j^t}{Q_i + c_i} \text{ and } c_{ii}(c_i) = p_j^t \frac{c_i}{Q_i + c_i}. \tag{13}$$

Using the QSS of $c_{ii} = c_{ii}(c_i)$ computed in (13), the QSS concentration of c_i can be found through the equality

$$F_i(c_i, \bar{c}_i) := \bar{c}_i - c_i - c_{ii}(c_i) = \bar{c}_i - c_i - p_j^t \frac{c_i}{Q_i + c_i} = 0. \tag{14}$$

For a fixed and bounded $\bar{c}_i > 0$, $F_i(c_i, \bar{c}_i)$ is a monotonically decreasing function of c_i and it satisfies $F_i(0, \bar{c}_i) = \bar{c}_i > 0$ and $F_i(+\infty, \bar{c}_i) = -\infty$. Thus, the equation $F_i(c_i, \bar{c}_i) = 0$ has a unique, positive solution $c_i = f_i(\bar{c}_i)$. In particular, this solution can be computed as:

$$c_i = f_i(\bar{c}_i) := \frac{1}{2}\left[(\bar{c}_i - p_j^t - Q_i) + \sqrt{(p_j^t + Q_i - \bar{c}_i)^2 + 4\bar{c}_i Q_i}\right]. \tag{15}$$

Substituting (15) into (13), we obtain the QSS concentration of p_j available for transcription:

$$p_j = p_j^t \frac{Q_i}{Q_i + f_i(\bar{c}_i)}. \tag{16}$$

Substituting (16) and (11) into (9) and let g be the vector representing the concentrations of all sgRNAs, the dynamics of g_i can be written as:

$$\frac{\mathrm{d}}{\mathrm{d}t} g_i = G_i(g) - (\delta + \theta_i) g_i - \delta \frac{D_T \cdot (g_i/K_i)}{1 + \sum_{j\in\mathcal{I}}(g_j/k_j)}, \tag{17}$$

where

$$G_i(g) = \begin{cases} u_i \cdot p_i^t, & \text{if } \mathrm{g}_i \text{ is not regulated by an sgRNA,} \\ \frac{u_i \cdot p_i^t \cdot Q_q}{Q_q + f_q(\bar{c}_q(g))}, & \text{if } \mathrm{g}_i \text{ is repressed by } \mathrm{g}_q, \end{cases} \quad \text{and} \quad \bar{c}_q(g) = \frac{D_T \cdot (g_q/K_q)}{1 + \sum_{k\in\mathcal{I}}(g_k/K_k)},$$

and function $f_q(\cdot)$ is defined as in (15).

dCas9 protein dynamics

The synthesis and decay of dCas9 protein (D) can be modeled by the chemical reactions:

$$\mathrm{p_D} \xrightarrow{\alpha_D} \mathrm{p_D} + \mathrm{D}, \qquad \mathrm{D} \xrightarrow{\delta} \varnothing, \tag{18}$$

where α_D is the synthesis rate constant of D from each copy of free promoter p_D driving dCas9 expression and δ is the dilution rate constant. The lumped parameter α_D increases with, for example, the dCas9's (i) promoter strength, (ii) plasmid copy number, and (iii) RBS strength. Based on mass action kinetics of the chemical reactions in (1), (2), and (18), we have

$$\frac{\mathrm{d}}{\mathrm{d}t} D = \alpha_D p_D - \delta D - \sum_{i\in\mathcal{I}}(a_i g_i D + d_i c_i) + \sum_{i\in\mathcal{I}_1} d_i c_{ii}. \tag{19}$$

Substituting the QSS concentrations of the complexes in (7) into (19), the free dCas9 dynamics can be written as:

$$\frac{\mathrm{d}}{\mathrm{d}t} D = \alpha_D p_D - \delta D \left(1 + \sum_{i\in\mathcal{I}} \frac{g_i}{K_i}\right). \tag{20}$$

The free promoter concentration p_D depends on whether dCas9 expression is regulated or not. Specifically, when dCas9 expression is unregulated, all promoters are available for transcription, and we set $p_D = p_D^t$. When dCas9 expression is repressed by sgRNA g_0, the promoter concentration satisfies the conservation law $p_D^t = p_D + c_{00}$. Similar to (16), using the QSS concentration of c_{00} derived in (7) and the relationship between $\bar{c}_0$ and c_0 derived in (15), we have

$$p_D^t = p_D + c_{00} = p_D\left(1 + \frac{c_0}{Q_0}\right), \qquad \Rightarrow \qquad p_D = \frac{p_D^t}{1 + c_0/Q_0} = p_D^t \frac{Q_0}{Q_0 + f_0(\bar{c}_0)}, \tag{21}$$

where p_D^t is the total concentration of promoter driving dCas9 expression and Q_0 is dissociation constant between complex c_0 and promoter p_D. Substituting (21) into (20), the free dCas9 concentration dynamics can be written as:

$$\frac{\mathrm{d}}{\mathrm{d}t}D = H(g) - \delta D\left(1 + \sum_{i\in\mathcal{I}}\frac{g_i}{K_i}\right), \tag{22}$$

where

$$H(g) = \begin{cases}\alpha_D \cdot p_D^t, & \text{if dCas9 unregulated,}\\ \frac{\alpha_D\cdot p_D^t\cdot Q_0}{Q_0+f_0(\bar{c}_0(g))}, & \text{if dCas9 is regulated,}\end{cases} \qquad \text{and} \qquad \bar{c}_0(g) = \frac{D_T\cdot(g_0/K_0)}{1+\sum_{i\in\mathcal{I}}(g_i/K_i)}.$$

The total concentration of dCas9 protein D_T is the summation of the concentration of apo-dCas9 D and the concentration of dCas9 proteins bound to sgRNAs:

$$D_T = D + \sum_{i\in\mathcal{I}_1}\bar{c}_i + \sum_{i\in\mathcal{I}_2}c_i, \qquad \frac{\mathrm{d}}{\mathrm{d}t}D_T = \frac{\mathrm{d}}{\mathrm{d}t}D + \sum_{i\in\mathcal{I}}\frac{\mathrm{d}}{\mathrm{d}t}c_i + \sum_{i\in\mathcal{I}_1}\frac{\mathrm{d}}{\mathrm{d}t}c_{ii}. \tag{23}$$

Hence, combining equations (5c)-(5e), and 20, we have the total dCas9 concentration dynamics:

$$\frac{\mathrm{d}}{\mathrm{d}t}D_T = \alpha_D p_D - \delta D_T = H(g) - \delta D_T. \tag{24}$$

Dynamics of other proteins

The circuit produces a set of proteins other than dCas9. Their production rates may depend on CRISPRi-based regulation. These proteins are denoted by y_i with index i taking values in set $\mathcal{I}_p$. The synthesis and dilution of protein y_i are governed by the following chemical reactions:

$$\mathrm{p_i} \xrightarrow{\alpha_i} \mathrm{p_i} + \mathrm{y_i}, \qquad \mathrm{y_i} \xrightarrow{\delta} \varnothing, \qquad i\in\mathcal{I}_p, \tag{25}$$

where α_i is the synthesis rate constant of y_i from each copy of free promoter p_i and δ is the dilution rate constant. Hence, by mass action kinetics, the dynamics of y_i can be written as

$$\frac{\mathrm{d}}{\mathrm{d}t}y_i = \alpha_i p_i - \delta y_i. \tag{26}$$

Transcription of y_i may be repressed by an sgRNA g_q. In particular, c_q (i.e., dCas9-sgRNA complex) may bind with p_i to form complex c_{qq}, prohibiting transcription. Following (7), the QSS concentration of c_{qq} is $c_{qq} = p_i c_q/K_q$. Since the copy number of DNA p_i is conserved, we have

$$p_i^t = p_i + c_{qq} = p_i\left(1+\frac{c_q}{Q_q}\right), \qquad \Rightarrow \qquad p_i = \frac{p_i^t}{1+c_q/Q_q} = p_i^t\frac{Q_q}{Q_q+f_q(\bar{c}_q)}, \tag{27}$$

where p_i^t is the total concentration of the promoter driving protein y_i expression and $f_q(\bar{c}_q)$ is defined as in (15). Substituting (27) into (26), the protein y_i dynamics can be written as:

$$\frac{\mathrm{d}}{\mathrm{d}t}y_i = P_i(g) - \delta y_i, \tag{28}$$

where

$$P_i(g) = \begin{cases}\alpha_i\cdot p_i^t, & \text{if } \mathrm{y}_i \text{ is constitutive,}\\ \frac{\alpha_i\cdot p_i^t\cdot Q_q}{Q_q+f_q(\bar{c}_q(g))}, & \text{if } \mathrm{y}_i \text{ is repressed by } \mathrm{g}_q,\end{cases} \qquad \text{and} \qquad \bar{c}_q(g) = \frac{D_T\cdot(g_q/K_q)}{1+\sum_{k\in\mathcal{I}}(g_k/K_k)}.$$

State variables	
c_i	concentration of dCas9-g_i complex
c_{ii}	concentration of dCas9-g_i-promoter complex
g_i	concentration of sgRNA g_i
D	concentration of apo-dCas9 protein
D_T	concentration of total dCas9 protein
y_i	concentration of protein i
**Parameters**	
p_i^t	total promoter concentration driving g_i or y_i production
p_D^t	total promoter concentration driving dCas9 protein production
u_i	sgRNA g_i synthesis rate constant from a single promoter
α_i	protein y_i synthesis rate constant from a single promoter
α_D	dCas9 synthesis rate constant from a single promoter
K_i	dissociation constant for sgRNA g_i and dCas9 binding
Q_i	dissociation constant for dCas9-g_i complex and target DNA binding
δ	dilution due to cell fission
θ_i	sgRNA g_i degradation rate constant

Supplementary Table 1: State variables and parameters invovled in the general CRISPRi-based circuit model (17), (24), and (28).

Summary

Supplementary Table 1 summarizes the state variables and parameters in the general CRISPRi-based circuit model in (17), (24), and (28). Based on these equations, the effects of dCas9 competition manifest in the following way. If g_j (or y_j) production is repressed by an sgRNA g_i ($i \neq j$), then the production rate of g_j (or y_j) is not only dependent on g_i, but also on the concentrations of all sgRNAs g in the circuit. This is because both functions $G_i(g)$ and $P_i(g)$ in (17) and (28) describing the production rates are g-dependent. To mitigate these unintended couplings arising from dCas9 competition, it is sufficient to maintain a constant level of apo-dCas9 concentration (D) that is independent of the concentration of competitor sgRNAs. Specifically, if D is a state-independent constant, then the concentrations of $\bar{c}_i$ and c_i in (7) depend only on g_i. As a consequence, p_j in (13) also depends only on g_i, and the sgRNA dynamic model can be re-written as:

$$\frac{\mathrm{d}}{\mathrm{d}t} g_i = G_i(g_q) - \left(\delta + \theta_i - \delta \frac{D}{K_i}\right) g_i, \tag{29}$$

where

$$G_i(g_q) = \begin{cases} u_i \cdot p_i^t, & \text{if } g_i \text{ is not regulated by an sgRNA,} \\ \frac{u_i \cdot p_i^t \cdot Q_q}{Q_q + f_q(\bar{c}_q(g_q))}, & \text{if } g_i \text{ is repressed by } g_q, \end{cases} \qquad \text{and} \qquad \bar{c}_q(g_q) = \frac{D g_q}{K_q},$$

which does not depend on sgRNAs other than the intended regulator g_q. Similarly, when apo-dCas9 level (D) is constant, the protein dynamics in (28) can be shown to depend only on the concentration of sgRNA repressing its promoter. We will show in Supplementary Note 1.3 that a practically constant D level can be achieved with the regulated dCas9 generator when the production rate of g_0, which represses dCas9 production, is sufficiently high.

Supplementary Note 1.2 Models of the NOT gate and the cascade

Here, we apply the general modeling framework developed in Supplementary Note 1.1 to the NOT gate and the cascade. Since dCas9 binds to a tract of the sgRNA that is the same for all the guides (tracr-region) and since the 20bp annealing with the target have been designed to achieve the maximum repression efficiency, we assume throughout this section that the dissociation constants between sgRNAs and dCas9 protein are

identical for all sgRNAs (i.e., $K_i = K$), that the dissociation constant between dCas9-sgRNA complex with their target promoters are identical (i.e., $Q_i = Q$), and that all sgRNAs have the same degradation rate constants (i.e., $\theta_i = \theta$). The main outcomes do not depend on these assumptions. Because of these assumptions, we can write $f_i(\cdot) = f(\cdot)$ for the function defined in (15).

NOT gate with unregulated dCas9 generator

The CRISPRi-based NOT gate in Figure 1 consists of two sgRNAs g_1 and g_2. sgRNA g_1 represses expression of the output protein RFP ($\mathrm{y} = \mathrm{y}_4$) and sgRNA g_2 does not have a DNA targeting site. Hence, we have $\mathcal{I} = \{1, 2\}$, $\mathcal{I}_1 = \{1\}$, $\mathcal{I}_2 = \{2\}$, and $\mathcal{I}_p = \{4\}$. Since the transcription of g_1 and g_2 are not regulated by other sgRNAs, using (17), their dynamics are:

$$\frac{\mathrm{d}}{\mathrm{d}t} g_1 = u_1 p_1^t - (\delta + \theta) g_1 - \delta \frac{D_T \cdot g_1}{K + g_1 + g_2}, \qquad \frac{\mathrm{d}}{\mathrm{d}t} g_2 = u_2 p_2^t - (\delta + \theta) g_2 - \delta \frac{D_T \cdot g_2}{K + g_1 + g_2}. \tag{30}$$

To model the fact that transcription of g_1 is HSL-inducible, the transcription rate of g_1 is modeled as a Hill function of HSL concentration: $u_1 = u_1(\mathrm{HSL})$. Mathematical expression of this Hill function can be found in equation (52) in Supplementary Note 1.4. RFP expression is repressed by g_1, hence, according to (28), we have

$$\frac{\mathrm{d}}{\mathrm{d}t} y_4 = \frac{\alpha_4 \cdot p_4^t \cdot Q}{Q + f(\bar{c}_1)} - \delta y_4, \qquad \text{where} \qquad \bar{c}_1 = \frac{D_T \cdot g_1}{K + g_1 + g_2}. \tag{31}$$

The total dCas9 concentration (D_T) dynamics follow (24) with $H(g) = \alpha_D p_D^t$, giving rise to

$$\frac{\mathrm{d}}{\mathrm{d}t} D_T = \alpha_D p_D^t - \delta D_T. \tag{32}$$

NOT gate with regulated dCas9 generator

For the NOT gate with regulated dCas9 generator, the circuit contains three sgRNA species, including g_0 that represses dCas9 expression. Hence, $\mathcal{I} = \{0, 1, 2\}$, $\mathcal{I}_1 = \{0, 1\}$, $\mathcal{I}_2 = \{2\}$, and $\mathcal{I}_p = \{4\}$. By (17), since g_1 and g_2 transcriptions are not regulated by other sgRNAs, we have

$$\begin{aligned} \frac{\mathrm{d}}{\mathrm{d}t} g_0 &= u_0 \cdot p_0^t - (\delta + \theta) g_0 - \delta \frac{D_T \cdot g_0}{K + g_0 + g_1 + g_2}, \\ \frac{\mathrm{d}}{\mathrm{d}t} g_1 &= u_1(\mathrm{HSL}) \cdot p_1^t - (\delta + \theta) g_1 - \delta \frac{D_T \cdot g_1}{K + g_0 + g_1 + g_2}, \\ \frac{\mathrm{d}}{\mathrm{d}t} g_2 &= u_2 \cdot p_2^t - (\delta + \theta) g_2 - \delta \frac{D_T \cdot g_2}{K + g_0 + g_1 + g_2}. \end{aligned} \tag{33}$$

According to (28), RFP expression dynamics follow:

$$\frac{\mathrm{d}}{\mathrm{d}t} y_4 = \frac{\alpha_4 \cdot p_4^t \cdot Q}{Q + f(\bar{c}_1)} - \delta y_4, \qquad \text{where} \qquad \bar{c}_1 = \frac{D_T \cdot g_1}{K + g_0 + g_1 + g_2}. \tag{34}$$

The dynamics of dCas9 protein are regulated and follow (24), giving rise to

$$\frac{\mathrm{d}}{\mathrm{d}t} D_T = \frac{\alpha_D \cdot p_D^t \cdot Q}{Q + f(\bar{c}_0(g))} - \delta D_T, \qquad \text{where} \qquad \bar{c}_0(g) = \frac{D_T \cdot g_0}{K + g_0 + g_1 + g_2}. \tag{35}$$

Cascade with unregulated dCas9 generator

The CRISPRi-based cascade shown in Figure 3 contains three sgRNAs. HSL-inducible g_1 represses transcription of sgRNA g_3, which subsequently represses expression of RFP y_4. sgRNA g_2 is transcribed constitutively

as a resource competitor. Hence, we have $\mathcal{I} = \{1,2,3\}$, $\mathcal{I}_1 = \{1,3\}$, $\mathcal{I}_2 = \{2\}$, and $\mathcal{I}_p = \{4\}$. According to (17), the sgRNA dynamics follow:

$$\begin{aligned}
\frac{\mathrm{d}}{\mathrm{d}t} g_1 &= u_1(\mathrm{HSL}) \cdot p_1^t - (\delta+\theta) g_1 - \delta \frac{D_T \cdot g_1}{K + g_1 + g_2 + g_3}, \\
\frac{\mathrm{d}}{\mathrm{d}t} g_2 &= u_2 \cdot p_2^t - (\delta+\theta) g_2 - \delta \frac{D_T \cdot g_2}{K + g_1 + g_2 + g_3}, \\
\frac{\mathrm{d}}{\mathrm{d}t} g_3 &= u_3 \cdot p_3^t \cdot \frac{Q}{Q + f(\bar{c}_1)} - (\delta+\theta) g_3 - \delta \frac{D_T \cdot g_3}{K + g_1 + g_2 + g_3},
\end{aligned} \tag{36}$$

where

$$\bar{c}_1 = \bar{c}_1(g_1) = \frac{D_T \cdot g_1}{K + g_1 + g_2 + g_3}.$$

The expression of RFP is repressed by g_3, hence, by (28), we have

$$\frac{\mathrm{d}}{\mathrm{d}t} y_4 = \frac{\alpha_4 \cdot p_4^t \cdot Q}{Q + f(\bar{c}_3)} - \delta y_4, \qquad \text{where} \qquad \bar{c}_3 = \frac{D_T \cdot g_3}{K + g_1 + g_2 + g_3}. \tag{37}$$

The total dCas9 concentration (D_T) dynamics are unregulated and follow (24), giving rise to

$$\frac{\mathrm{d}}{\mathrm{d}t} D_T = \alpha_D p_D^t - \delta D_T. \tag{38}$$

Cascade with regulated dCas9 generator

For the cascade circuit with regulated dCas9 generator, we take into account the additional sgRNA g_0 to repress expression of dCas9. Hence, in this system, we have $\mathcal{I} = \{0,1,2,3\}$, $\mathcal{I}_1 = \{0,1,3\}$, $\mathcal{I}_2 = \{2\}$, and $\mathcal{I}_p = \{4\}$. By (17), the sgRNA dynamics are:

$$\begin{aligned}
\frac{\mathrm{d}}{\mathrm{d}t} g_0 &= u_0 \cdot p_0^t - (\delta+\theta) g_0 - \delta \frac{D_T \cdot g_0}{K + g_0 + g_1 + g_2 + g_3}, \\
\frac{\mathrm{d}}{\mathrm{d}t} g_1 &= u_1(\mathrm{HSL}) \cdot p_1^t - (\delta+\theta) g_1 - \delta \frac{D_T \cdot g_1}{K + g_0 + g_1 + g_2 + g_3}, \\
\frac{\mathrm{d}}{\mathrm{d}t} g_2 &= u_2 \cdot p_2^t - (\delta+\theta) g_2 - \delta \frac{D_T \cdot g_2}{K + g_0 + g_1 + g_2 + g_3}, \\
\frac{\mathrm{d}}{\mathrm{d}t} g_3 &= u_3 \cdot p_3^t \cdot \frac{Q}{Q + f(\bar{c}_1)} - (\delta+\theta) g_3 - \delta \frac{D_T \cdot g_3}{K + g_0 + g_1 + g_2 + g_3},
\end{aligned} \tag{39}$$

where

$$\bar{c}_1 = \bar{c}_1(g_1) = \frac{D_T \cdot g_1}{K + g_0 + g_1 + g_2 + g_3}.$$

According to (28), the dynamics of RFP expression can be written as:

$$\frac{\mathrm{d}}{\mathrm{d}t} y_4 = \frac{\alpha_4 \cdot p_4^t \cdot Q}{Q + f(\bar{c}_3)} - \delta y_4, \qquad \text{where} \qquad \bar{c}_3 = \frac{D_T \cdot g_3}{K + g_1 + g_2 + g_3}. \tag{40}$$

The total dCas9 concentration (D_T) dynamics are regulated and follow (24), giving rise to:

$$\frac{\mathrm{d}}{\mathrm{d}t} D_T = \frac{\alpha_D \cdot p_D^t \cdot Q}{Q + f(\bar{c}_0(g))} - \delta D_T, \qquad \text{where} \qquad \bar{c}_0(g) = \frac{D_T \cdot g_0}{K + g_0 + g_1 + g_2 + g_3}. \tag{41}$$

Supplementary Note 1.3 Model guided design of the regulated dCas9 generator

In this section, we consider the regulated dCas9 generator and demonstrate that increasing sufficiently the synthesis rate constant of g_0 (i.e., u_0) increases the robustness of a CRISPRi-NOT gate to the presence of a competitor sgRNA (i.e., g_2). In particular, our analysis indicates that the sensitivity of apo-dCas9 concentration (D) to competitor sgRNA DNA copy number (p_2^t) can be made arbitrarily small by increasing u_0.

Sensitivity of apo-dCas9 concentration to competitor sgRNA

To be consistent with our notation in the main text, in addition to g_2, sgRNA g_0 represses dCas9 expression and g_1 represses the output. The free sgRNA concentrations g_i ($i = 0, 1, 2$) depends on D. Specifically, at steady state, by setting the time derivative in (17) to zero, for $i = 0, 1, 2$, we obtain:

$$0 = u_i p_i^t - (\delta + \theta_i) g_i - \delta \frac{D_T \cdot (g_i/K_i)}{1 + \sum_{j=0,1,2} (g_j/k_j)} = u_i p_i^t - (\delta + \theta_i) g_i - \delta D \frac{g_i}{K_i}, \tag{42}$$

where we use $D = D_T/(1 + \sum_{i \in \mathcal{I}} (g_i/K_i))$ in (10) to attain the last equality. By (42), the steady state g_i satisfies:

$$g_i = g_i(D) = \frac{u_i p_i^t}{\delta + \theta_i + \delta D/K_i}, \; i = 0, 1, 2. \tag{43}$$

Since we study the system's performance with $p_2^t = 0$ and $p_2^t \neq 0$, we specifically write $g_2 = g_2(D, p_2^t)$. By setting the time derivative in (20) to zero, the steady state concentration of D can be solved from:

$$\begin{aligned} \phi(D, p_2^t) :&= \alpha_D p_D^t \frac{Q_0}{Q_0 + f_0(\bar{c}_0)} - \delta D \left(1 + \frac{g_0(D)}{K_0} + \frac{g_1(D)}{K_1} + \frac{g_2(D, p_2^t)}{K_2}\right) \\ &= \alpha_D p_D^t \frac{Q_0}{Q_0 + f_0\left(\frac{D g_0(D)}{K_0}\right)} - \delta D \left(1 + \frac{g_0(D)}{K_0} + \frac{g_1(D)}{K_1} + \frac{g_2(D, p_2^t)}{K_2}\right) = 0, \end{aligned} \tag{44}$$

where we use $\bar{c}_0 = D g_0/K_0$ dervied in (7) in the last equality. The relative sensitivity of D to p_2^t for the regulated dCas9 generator, which we denote by $\mathcal{S}_{\mathrm{R}}$, can be computed from (44) as:

$$\mathcal{S}_{\mathrm{R}} := \frac{1}{D} \cdot \left| \frac{\mathrm{d}D}{\mathrm{d}p_2^t} \right| = \frac{1}{D} \cdot \frac{|\partial \phi / \partial p_2^t|}{|\partial \phi / \partial D|}, \tag{45}$$

where we use the equality

$$\frac{\mathrm{d}}{\mathrm{d}p_2^t} D = -\frac{\partial \phi / \partial p_2^t}{\partial \phi / \partial D}$$

according to the implicit function theorem [S2]. From (43) and (44), we find

$$\begin{aligned} \frac{\partial \phi}{\partial p_2^t} &= -\frac{\delta D}{K_2} \cdot \frac{u_2}{\delta + \theta_2 + \delta D/K_2}, \\ \frac{\partial \phi}{\partial D} &= -\alpha_D p_D^t Q_0 \cdot \frac{\mathrm{d} f_0 / \mathrm{d}\bar{c}_0}{[Q_0 + f_0(\bar{c}_0)]^2} \cdot \frac{g_0}{K_0} - \delta \left[1 + \sum_{i=0,1,2} \frac{1}{K_i} \frac{\mathrm{d}}{\mathrm{d}D} (D g_i(D)) \right]. \end{aligned} \tag{46}$$

From (43), for $i = 0, 1, 2$, we obtain

$$D g_i(D) = \frac{D u_i p_i^t}{\delta + \theta_i + \delta D/K_i}, \qquad \Rightarrow \qquad \frac{\mathrm{d}}{\mathrm{d}D} (D g_i) = u_i p_i^t \frac{\delta + \theta_i}{(\delta + \theta_i + D/K_i)^2}. \tag{47}$$

Substituting $0 \le D \le D_T \le \alpha_D p_D^t/\delta$ into (46) and (47), we find

$$\left|\frac{\partial \phi}{\partial p_2^t}\right| \le \frac{\delta D}{K_2}\cdot\frac{u_2}{\delta+\theta_2}, \qquad \text{and} \qquad \frac{u_i p_i^t}{\delta+\theta_i} \ge \frac{\mathrm{d}}{\mathrm{d}D}(Dg_i) \ge \frac{u_i p_i^t(\delta+\theta_i)}{(\delta+\theta_i+\alpha_D p_D^t/(K_i\delta))^2}. \tag{48}$$

On the other hand, by the definition of f_0 in (15), we have $\mathrm{d}f_0/\mathrm{d}\bar{c}_0 > 0$ for all $\bar{c}_0$. Combining this fact with the inequality in (48), we can find a lower bound for $|\partial\phi/\partial D|$ in (46):

$$\left|\frac{\partial \phi}{\partial D}\right| > \frac{\delta}{K_0}\cdot\frac{\mathrm{d}}{\mathrm{d}D}(Dg_0) \ge \frac{\delta u_0 p_0^t(\delta+\theta_0)}{K_0[\delta+\theta_0+\alpha_D p_D^t/(K_0\delta)]^2}. \tag{49}$$

Substituting (48) and (49) into (45), and suppose that $K_i, p_i^t.p_D^t, \delta, \theta_i, u_1, u_2$, and α_D are all positive constants, we can find an upper bound for $\mathcal{S}_\mathrm{R}$ that depends on u_0:

$$\mathcal{S}_\mathrm{R} = \mathcal{S}_\mathrm{R}(u_0) < \frac{u_2}{\delta+\theta_2}\cdot\frac{[\delta+\theta_0+\alpha_D p_D^t/(K_0\delta)]^2}{u_0 p_0^t(\delta+\theta_0)}\cdot\frac{K_0}{K_2} =: \bar{\mathcal{S}}_\mathrm{R}(u_0). \tag{50}$$

According to (50), the sensitivity upper bound $\bar{\mathcal{S}}_\mathrm{R}$ is a monotonically decreasing function of the g_0 production rate constant u_0. Additionally, $\lim_{u_0\to\infty}\bar{\mathcal{S}}_\mathrm{R}(u_0) = 0$. Because $\mathcal{S}_\mathrm{R}(u_0) \ge 0$ and $\mathcal{S}_\mathrm{R}(u_0) < \bar{\mathcal{S}}_\mathrm{R}(u_0)$, (50) implies that $\lim_{u_0\to\infty}\mathcal{S}_\mathrm{R}(u_0) = 0$. Hence, for a regulated NOT gate, if u_0 is sufficiently large, then the apo-dCas9 concentration D becomes insensitive to the presence of g_2 DNA (i.e., p_2^t). We verify this model prediction through simulations. In Supplementary Figure 1, we simulate the dose response curves of CRISPRi-based NOT gates with different g_0 synthesis rate constants u_0 and dCas9 protein synthesis rate constants α_D. We find that as shown by our analysis, for each fixed α_D, the dose response curves becomes independent of g_2 when u_0 is sufficiently large.

Physically, this increase in robustness is due to the presence of g_0 that creates negative feedback actions on free dCas9 (D) dynamics. In particular, according to (22):

$$\frac{\mathrm{d}}{\mathrm{d}t}D = \underbrace{\frac{\alpha_D p_D^t Q_0}{Q_0+f_0(Dg_0/K_0)}}_{\text{(I)}} - \delta D\left(1+\underbrace{\frac{g_0}{K_0}}_{\text{(II)}}+\sum_{i\neq 0}\frac{g_i}{K_i}\right), \tag{51}$$

in which the feedback actions take two forms. On the one hand, with reference to the term labeled (I), a decrease in D leads to a decrease in $f_0(Dg_0/K_0)$ to increase the production rate of D. On the other hand, with reference to the term labeled (II) in equation, a drop in D also results in a decreased effective decay rate of D. Both forms of feedback actions contribute to the decrease in sensitivity of D to g_2. Specifically, as we derive in (45) and (49), a small $\mathcal{S}_\mathrm{R}$ is due to a large $|\partial\phi/\partial D|$, which is computed in (46). The feedback effect arising from dCas9 production rate change is manifested in the first term in (46), while the feedback effect arising from dCas9 effective decay rate change is manifested in the term encompassing $\mathrm{d}(Dg_0)/\mathrm{d}D$ in (46). Increasing the magnitude of both terms contributes to an increase in $|\partial\phi/\partial D|$, hence, a decrease in $\mathcal{S}_\mathrm{R}$. Therefore, both physical forms of feedback increase robustness of D to g_2. As it can be observed from (51), increasing g_0 (via, for example, increasing u_0) can induce larger effects from both forms of feedback, which is consistent with our analysis in (50).

Experimental validation of sensitivity analysis

We designed an experiment to verify that sufficiently increasing u_0 decreases $\mathcal{S}_\mathrm{R}$. In particular, as shown in Supplementary Figure 5 (left and middle panels), we tested and compared two regulated dCas9 generators with same promoters and RBS for dCas9 production but with different promoters driving g_0 production, which give rise to different u_0 parameters. The two regulated dCas9 generators were co-transformed with the CRISPRi-NOT gate into *E. coli* NEB10B strain. The NOT gate either contains no competitor sgRNA or a competitor sgRNA g_2 driven by the BBa_J23100 promoter. For the regulated dCas9 generator with g_0 driven by the weaker BBa_J23116 promoter, the dose-response curve of the NOT gate is highly sensitive to

the presence of g_2. In fact, with reference to Supplementary Figure 5b (left panel), for low HSL levels, the fold change in RFP expression due to g_2 production is similar to that induced by g_2 when dCas9 production is unregulated (Figure 1c). On the other hand, with reference to Supplementary Figure 5b (middle panel), when the dCas9 generator is regulated by g_0 driven by the stronger P108 promoter, RFP expression becomes insensitive to g_2, indicating that robustness of apo-dCas9 concentration to competitor sgRNA production can indeed be achieved when the synthesis rate constant of g_0 is sufficiently high.

Increasing dCas9 synthesis rate constant to maintain fold repression

Increasing g_0 synthesis rate constant also increases repression on dCas9 synthesis, resulting in reduced total dCas9 concentration, hence reducing the concentration of dCas9-sgRNA complexes to repress target promoters. In fact, with reference to Supplementary Figure 1, for regulated dCas9 generators with small dCas9 synthesis rates (α_D), increasing u_0 leads to significant reductions in the fold repression of the NOT gates' outputs. In order to achieve robustness to competitor sgRNA while maintaining fold repression by the gates' sgRNAs, one can increase dCas9 synthesis rate in the regulated generator. This can be achieved by increasing the promoter strength driving the expression of dCas9 and/or the RBS strength of dCas9 transcript. In Supplementary Figure 1, our simulations indicate that when u_0 is large, increasing dCas9 synthesis rate constant α_D does not decrease robustness to competitor sgRNA. This result is further supported by our experiments in Supplementary Figure 5 (middle and right side panels). With reference to Supplementary Figure 5a, the regulated dCas9 generator in these two panels have identical u_0 but different synthesis rate constants α_D for dCas9 protein. In particular, while dCas9 expressions in both systems are driven by P104 promoters, the RBS strength of the system in the right panel is $\sim$ 3000x stronger than that of the system in the middle panel, indicting a $\sim$ 3000x increase in α_D. While in both systems, the competition effects by sgRNA g_2 can be almost entirely mitigated by the regulator, the output of the system in the right panel shows larger fold repression. This difference is most significant when HSL=1 nM, where output level of the system with smaller α_D is about 3x larger than that of the system with larger α_D. Hence, based on these simulations and experiments, in order to increase robustness of CRISPRi-based circuits to dCas9 competition while maintaining similar fold repression, in the regulated dCas9 generator, we choose to transcribe g_0 from a strong promoter (P112) while at the same time also express dCas9 protein from a stronger promoter (P104) using a stronger RBS.

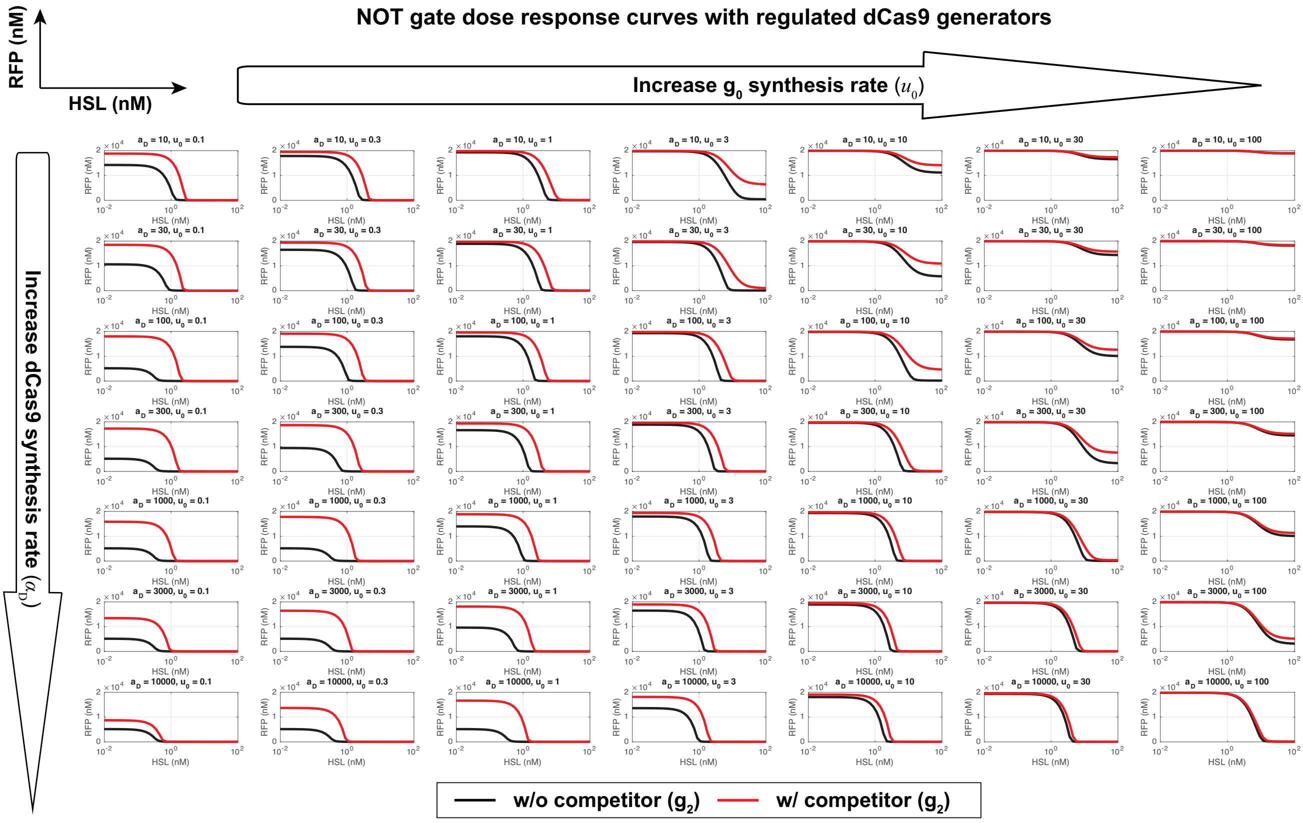

Supplementary Figure 1: **Dose response curves of the NOT gate with different regulated dCas9 generators.** The generators have different dCas9 synthesis rate constants (α_D) and g_0 synthesis rate constants (u_0). Black and red dose response curves correspond to systems without and with the competitor sgRNA g_2, respectively. Model and parameters for simulations can be found in Supplementary Table 2 and Supplementary Table 3, respectively.

Effects of changing K on competition

It is known from *in vitro* studies that the value of the effective binding rate constant of dCas9 with sgRNA may be subject to large variation due to non-specific binding of dCas9 with RNA [S3]. We therefore investigated the effect of changing the dissociation constants K and Q on the steady state characteristics given that they are inversely proportional to the binding rate constant (see equation (8) in Supplementary Note 1.1). Simulations show no appreciable effect of changes in K and Q as shown in the two following figures.

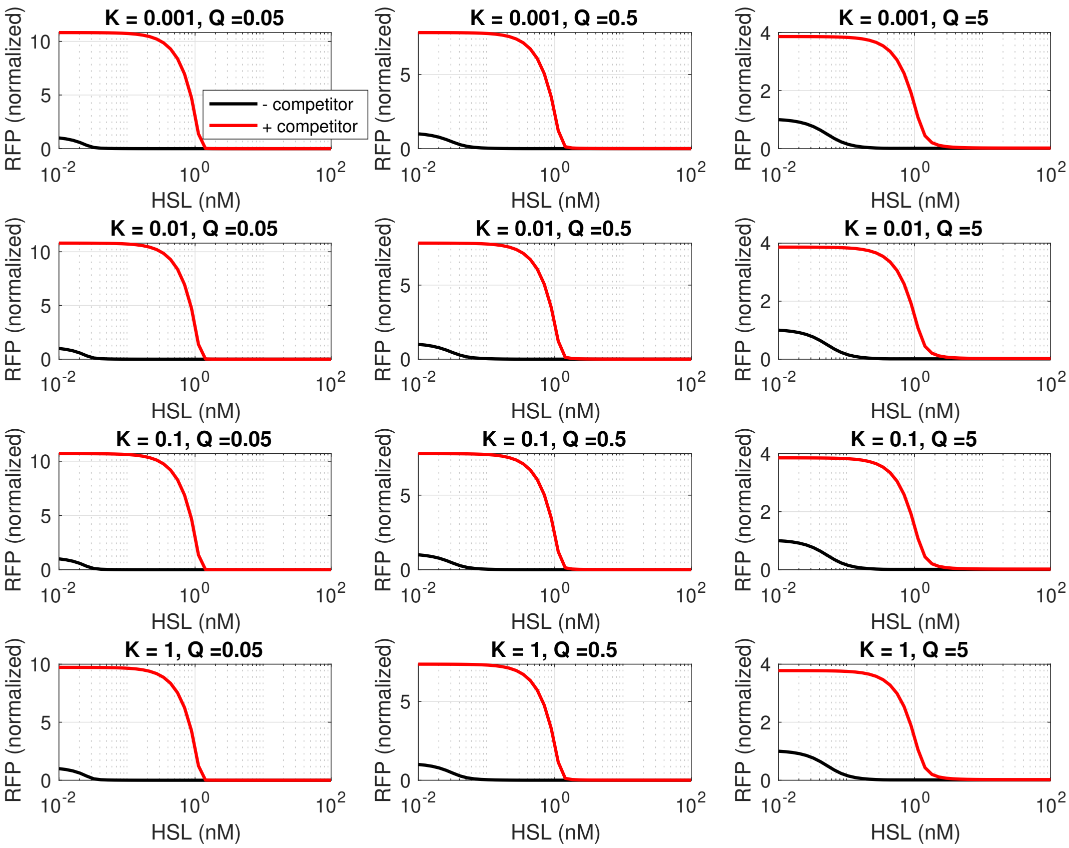

Supplementary Figure 2: **NOT gate I/O steady state characteristic with unregulated dCas9 generator for different values of the dissociation constants between gRNA and dCas9 (K) and of the dissociation constants between gRNA:dCas9 complex with DNA (Q).** The presence of competitor gRNA leads to slightly different dose response curves of the NOT gate when K is changed. All parameters except for K and Q are identical to those for the high copy NOT gate reported in Supplementary Table 3, Supplementary Table 4 and Supplementary Table 13

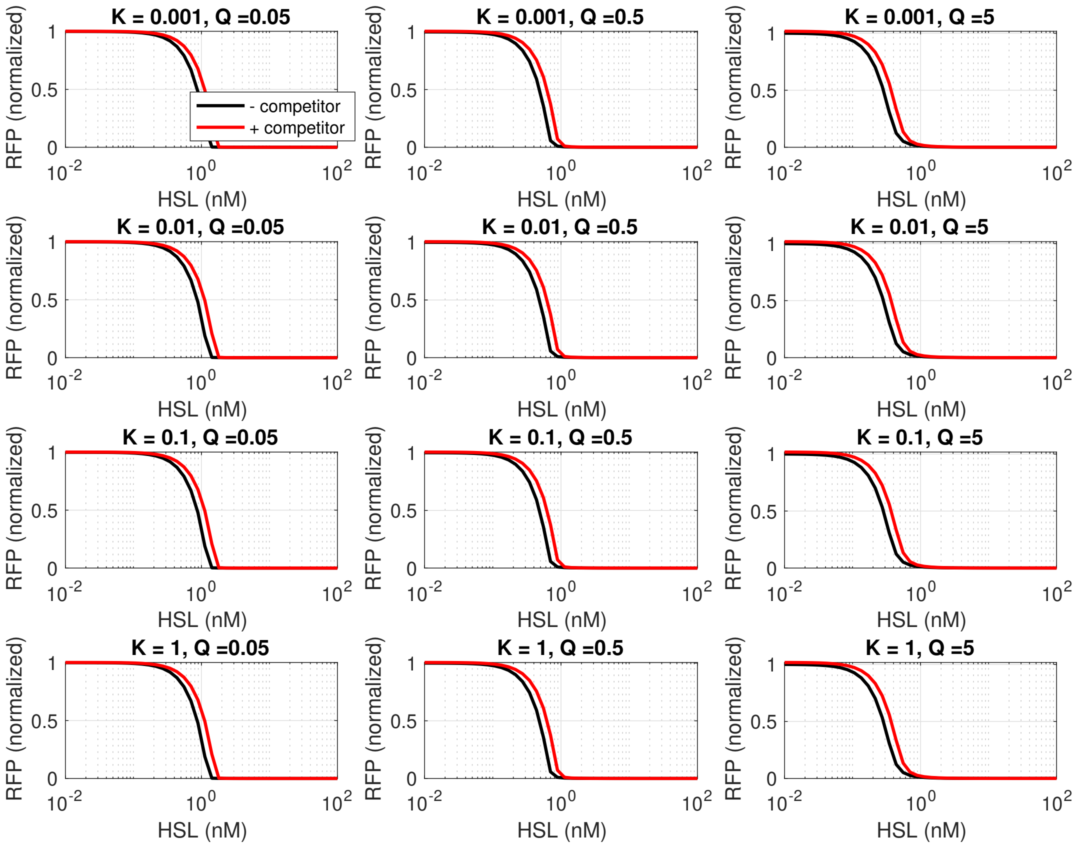

Supplementary Figure 3: **NOT gate I/O steady state characteristic with regulated dCas9 generator for different values of the dissociation constants between gRNA and dCas9 (K) and of the dissociation constants between gRNA:dCas9 complex with DNA (Q).** Robustness of the NOT gate to the presence of competitor gRNA is essentially independent of dissociation constants K and Q. All parameters except K and Q are identical to those for the high copy NOT gate reported in Supplementary Table 3, Supplementary Table 4 and Supplementary Table 13.

Supplementary Note 1.4 Numerical Simulations

Numerical simulations were carried out using MATLAB R2020a with variable step ODE solver `ode15s` to obtain the simulation results in Figures 2-3 and Supplementary Figure 1-Supplementary Figure 3. In particular, the equations used for simulations are summarized in Supplementary Table 2.

Circuit	Figure	Equations
NOT gate, unregulated dCas9 generator	Fig. 2a,c, Supp. Fig. 1-3	(30), (31), (32)
NOT gate, regulated dCas9 generator	Fig. 2b,d Supp. Fig. 1-3	(33), (34), (35)
cascade, unregulated dCas9 generator	Fig. 3c	(36), (37), (38)
cascade, regulated dCas9 generator	Fig. 3d	(39), (40), (41)

Supplementary Table 2: Equations used for simulations.

The parameters used for the simulations are listed in Supplementary Table 3. To obtain the plasmid concentrations, we follow the standard assumption that 1 copy/cell = 1 nM in bacteria *E. coli* [S4]. We use identical NOT gate parameters for simulations in the main text (Figure 2) and in Supplementary Figure 1-Supplementary Figure 3.

The protein dilution rate constant δ is set to the average *E. coli* doubling time found in our experimental conditions. Since neither dCas9 nor RFP is targeted by a protease, we assume that protein degradation is negligible. The sgRNA synthesis rates u_i for $i = 0, 1, 2, 3$ are set to match the experimental qualitative I/O responses reported in Figure 2 and Figure 3. The rank of the magnitudes of the synthesis rates matches the promoter strength rank in Supplementary Table 13. The increase in dCas9 synthesis rate in the regulated dCas9 generator reflects the design choice that dCas9 promoter and RBS are both much stronger in the regulated generator. Synthesis rate of g_1 is modulated by the concentration of HSL. Hence, we use the following Hill function to model its synthesis rate u_1:

$$u_1 = V_{\max} \cdot \left[\delta_0 + \frac{(\mathrm{HSL}/k_{\mathrm{eff}})^\eta}{(\mathrm{HSL}/k_{\mathrm{eff}})^\eta + 1}\right], \qquad \text{where,} \qquad k_{\mathrm{eff}} := k_d \cdot \frac{k}{\mathrm{LuxR}}, \tag{52}$$

$V_{\max}$ is the maximum synthesis rate from the pLux promoter, k is the dissociation constant between HSL and LuxR protein, k_d is the dissociation constant between HSL-LuxR complex and pLux promoter, η is the Hill coefficient, and δ_0 represents the leakiness from the pLux promoter. We use the following parameters for equation (52) in simulations. The effective dissociation constant k_{eff} is smaller for the cascade system because LuxR protein is encoded on a higher copy plasmid there. Consequently, the concentration of LuxR is higher in the cascade system, leading to a reduced k_{eff}.

**Para.**	**Unit**	**Fig. 2b**	**Fig. 2c**	**Fig. 2d**	**Fig. 2e**	**Fig. 3c**	**Fig. 3d**	**Supp. Fig. 1**	**Supp. Fig. 2**	**Supp. Fig. 3**	**Ref.**
u_2		0.3 (low)		5 (low)		40		20	40		-
		40 (high)		20 (high)							
u_3		-				24		-	-		-
u_0	[min^{-1}]	65						varies	65		-
α_D		0.6	6000	0.6	6000	0.6	6000	varies	0.6	6000	-
α_4		1									-
δ		0.01									exp.
θ		0.2									[S5]
p_0^t		30									[S6]
$p_1^t = p_2^t$		84		5		84		5	84		
p_3^t		-				84		-			
p_4^t	[nM]	84		200		84		200	84		
p_D^t		30									
K		0.01							varies		[S7]
Q		0.5							varies		[S8]

Supplementary Table 3: Parameters used for simulations.

**Parameter**	V_{max} [min^{-1}]	k_{eff} [nM]	η	δ_0
**NOT gate (LC)**	50	8	2	6E-3
**NOT gate (HC)**	50	6	2	1.8E-4
**Cascade**	50	5	2	6E-3

Supplementary Table 4: Values of parameters describing Hill activation for g_1 synthesis

Supplementary Note 2

Overcoming dCas9 toxicity during pdCas9_CL transformation

Overexpression of dCas9, even in transient, non-steady state fashion, can be toxic to the cell [S9]. Transient expression of dCas9 when transforming plasmid pdCas9_CL, which encodes the regulated dCas9 generator, leads to severe toxicity. This is due to high dCas9 protein expression driven by a strong promoter (Supplementary Table 13) and to the fact that right after transformation there is not a sufficient amount of dCas9-g_0 complex to repress this strong promoter. In fact, immediately after this plasmid is transformed into a cell, the initial concentrations of sgRNA g_0 and dCas9 are both zero, but dCas9 production rate is high and, due to a zero g_0, is unrepressed. Thus, dCas9 concentration increases rapidly after the plasmid is transformed and before g_0 level is sufficiently high to repress dCas9 transcription. This, in turn, may create an overshoot in dCas9 concentration resulting in toxic effects to cells. This overshoot in dCas9 level may explain the cell death observed during transformation in our experiments.

To decrease the overshoot in dCas9 concentration following transformation of the plasmid, we prepared a host cell strain encompassing a removable module that can repress dCas9 expression once the pdCas9_CL plasmid is transformed into the host cell. Specifically, this removable module is a temperature-sensitive plasmid pAUX-OL (Supplementary Figure 4) that expresses both dCas9 and g_0 at low levels (Supplementary Table 13). This way, when pdCas9_CL is transformed in cells, there is already a basal level of the repressive dCas9-g_0 complex that represses the strong promoter driving dCas9 transcription in the pdCas9_CL plasmid. The pAUX-OL plasmid is removed subsequently through plasmid curing at 42 °C by virtue of the temperature-sensitive origin of replication. Specifically, plasmid pAUX-OL was constructed by taking, as a plasmid backbone, a sequence from the pKD46 plasmid [S10] comprising the origin of replication with a temperature-sensitive replication initiation and an ampicillin resistance cassette. The plasmid contains the sgRNA expression cassette from pdCas9_CL and the dCas9 expression cassette from pdCas9_OP, assembled in a three-part Gibson Assembly. The plasmid map can be found in Supplementary Figure 4.

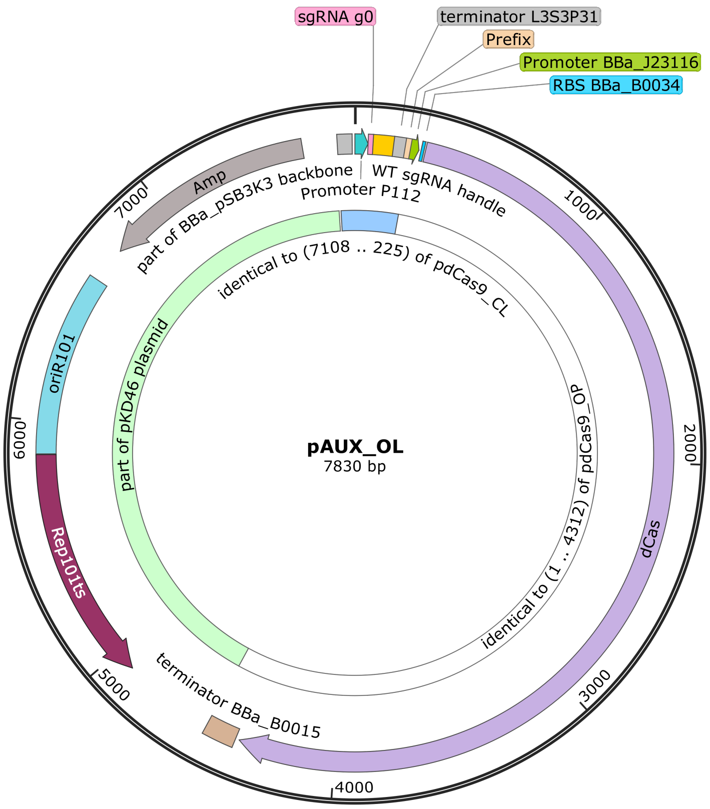

Supplementary Figure 4: **Map of the plasmid pAUX_OL.** The three major fragments for the assembly are annotated in the inner ring of the map. Specifically, the sgRNA g0 transcription cassette was cloned from pdCas9_CL plasmid. The dCas9 gene cassette driven by BBa_J23116 promoter is from pdCas9_OP plasmid (Supplementary Figure 9). The origin of replication, temperature-sensitive replication initiation protein and ampicillin resistance cassette is from plasmid pKD46 [S10].

Supplementary Note 3

Effect of sgRNA g0 transcription level on robustness of regulated dCas9 generators

To demonstrate the effect of sgRNA g0 transcription level on robustness of regulated dCas9 generators, we prepared two regulated dCas9 generators capable of transcribing different amounts of sgRNA g0. Specifically, we used the weak BBa_J23116 and the strong P112 promoters to transcribe sgRNA g0 of the regulated dCas9 generators in the pdCas9_CL2 and pdCas9_CL7 plasmids, respectively (Supplementary Table 5). The ribosome binding sites of *dCas9* genes in both plasmids are the BBa_B0034 RBS. The pdCas9_CL2 and pdCas9_CL7 plasmids were co-transformed with the same plasmids of the plasmids 2 and 3 used in Supplementary Table 10 to create the constructs pCL92, pCL 94, pCL96 and pCL98 (see Supplementary Table 5) in order to compare to the constructs pCL87 and pCL89 (see Supplementary Table 10), respectively. The genetic diagrams of pCL96 and pCL98 are shown in Supplementary Figure 5a (the left and middle panels, respectively). We aim to compare how the dose-response curves of the CRISPRi-based NOT gate will change in the absence and the presence of the competitor sgRNA when apo-dCas9 proteins are expressed from the regulated dCas9 generator which transcribes sgRNA g0 in either low or high level.

Indeed, the response of the NOT gate is significantly affected by the competitor sgRNA at low HSL induction levels when the regulated dCas9 generator transcribes sgRNA g0 with the weak BBa_J23116 promoter as shown in Supplementary Figure 5b (the left panel). The fold-change can be up to 2-fold at the given level of the competitor sgRNA (Supplementary Figure 5c (the left panel)). This extent of the fold-change is similar to the one observed with the unregulated dCas9 generator (Figure 2b), suggesting that this regulated dCas9 generator dose not mitigate dCas9 competition because of low sgRNA g0 transcription. On the contrary, the response of the NOT gate is independent of the presence of the competitor sgRNA when the regulated dCas9 generator transcribes sgRNA g0 with the strong P112 promoter as shown in Supplementary Figure 5b (the middle panel). The fold-change remains practically unity (Supplementary Figure 5c (the middle panel)). The specific growth rates are similar among different constructs across the induced HSL concentrations as 0, 0.001, 0.01, 0.1, 1, and 50 nM (Supplementary Figure 5d, the left and middle panels). The experimental data are in agreement with the sensitivity analysis of the regulated dCas9 generator (Supplementary Note 1.3) such that increasing the synthesis rate of sgRNA g0 increases the robustness of a CRISPRi-based NOT gate to the presence of a competitor sgRNA.

Effect of dCas9 production rate on fold repression of CRISPRi-based NOT gate

When the ribosome binding site of *dCas9* gene is changed from the BBa_B0034 to the RBS1, comparing the genetic diagram of Supplementary Figure 5a in the middle panel to the one in the right-side panel, the predicted translation initiation rate (TIR) is changed from 443 to 1384771. This change significantly increase dCas9 production rate in the regulated dCas9 generator. The RBS1 was designed to achieve the predicted maximal TIR by RBS calculator 2.0 [S11]. The fold-changes remain almost the same as shown in the middle and right-side panels of Supplementary Figure 5c, but the dose-response curves, especially at 1 nM HSL, show a better repression on the CRISPRi target in the right panel than in the middle panel of Supplementary Figure 5b. This suggests that higher dCas9 production rate does not affect the robustness of the regulated dCas9 generator but improves fold repression of the CRISPRi-based NOT gate. Similar specific growth rates of different constructs across the induced HSL concentrations, as shown in Supplementary Figure 5d, suggest that increased dCas9 level did not lead to any cytotoxicity. The experimental data are in agreement with the analysis of Supplementary Note 1.3 and the simulations of Supplementary Figure 1, according to which the production rate of dCas9 can be increased to improve the fold repression of CRISPRi while keeping robustness if the sgRNA g0 production rate is sufficiently large.

[1] We created the pdCas9_CL2 plasmid by changing the promoter of sgRNA g0 in the pdCas9_CL (refer to the plasmid map Supplementary Figure 9b) from the P112 promoter to the BBa_J23116 promoter.

[2] The plasmids 2 and 3 are from the same set of the plasmids 2 and 3 in Supplementary Table 10.

construct	plasmid 1 (Kan)[1]	plasmid 2 (Amp)[2]	plasmid 3 (Cm)	sgRNA g0's promoter	sgRNA g2's promoter
pCL92	pdCas9_CL2	I13521-target	AEgPtet_No_competitor	BBa_J23116	cassette absent
pCL94	pdCas9_CL7	I13521-target	AEgPtet_No_competitor	P112	cassette absent
pCL96	pdCas9_CL2	I13521-target	AEgPtet_100gPlac	BBa_J23116	BBa_J23100
pCL98	pdCas9_CL7	I13521-target	AEgPtet_100gPlac	P112	BBa_J23100

Supplementary Table 5: List of the constructs used in Supplementary Figure 5. Each construct is obtained from co-transforming the indicated plasmids into *E. coli* NEB10B strain.

.

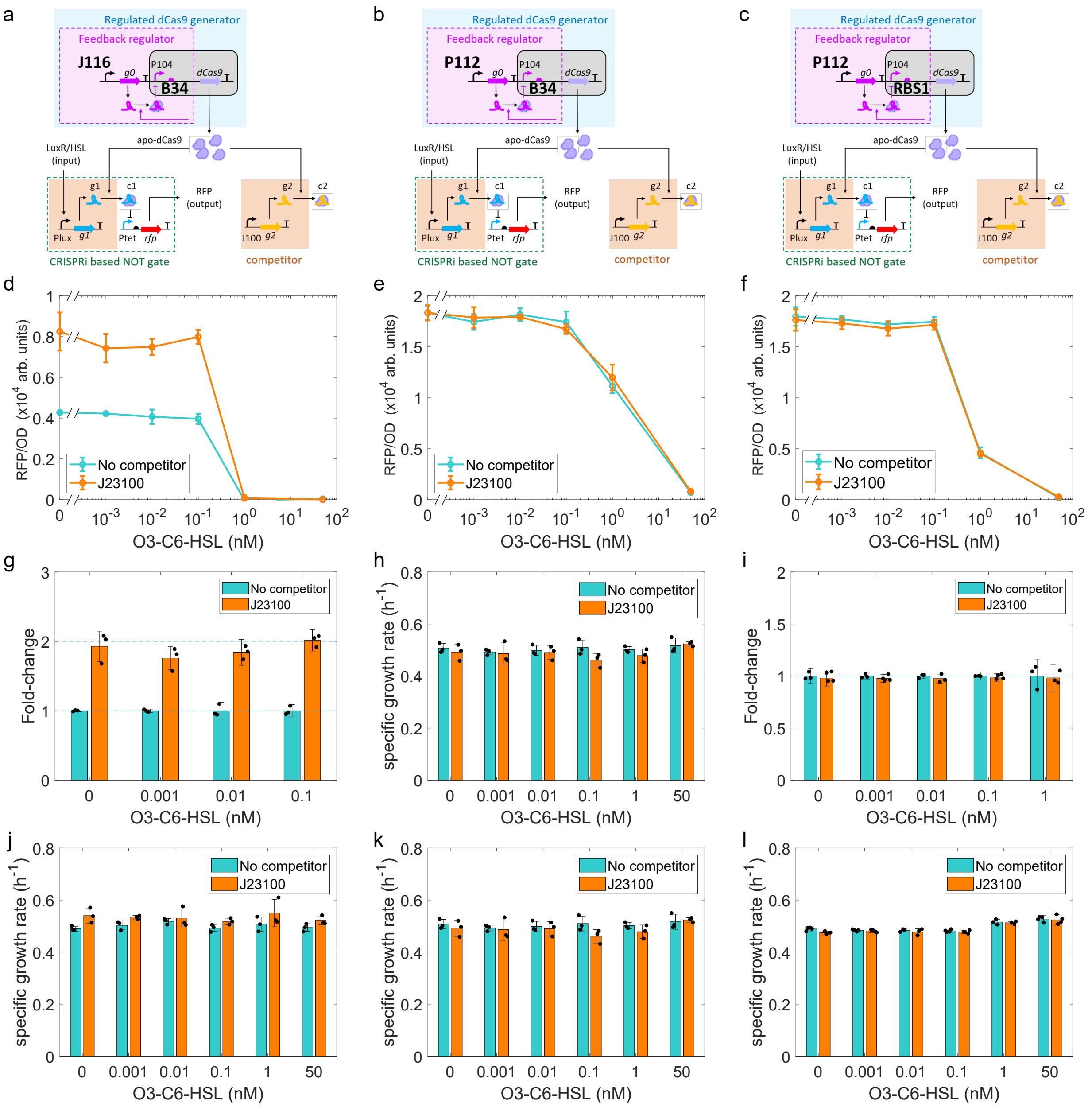

Supplementary Figure 5: **Abundant sgRNA g0 contributes to robustness of regulated dCas9 generator and higher dCas9 production rate improves fold repression.** Apo-dCas9 proteins are expressed by the regulated dCas9 generator in which the promoter of sgRNA g0 and the ribosome binding site of *dCas9* gene are in a pair as (promoter, RBS) as (BBa_J23116, BBa_B0034), (P112, BBa_B0034), and (P112, RBS1) in the left, middle, and right-side panels, respectively. (a-c) Genetic circuits of the CRISPRi-based NOT gates and the competitor modules are identical to the one used in Figure 1 except for the promoter driving g_0 expression. The competitor module is either absent or using the BBa_J23100 promoter to transcribe the competitor sgRNA g2. (d-f) Comparison of dose-response curves in the absence or presence of the competitor module. (g-i) Fold-changes at a given HSL induction were computed as described in Methods section Quantification of competition effects, by dividing the RFP/OD value of a construct by the one of the construct lacking the competitor module. (j-l) Specific growth rate of each construct at a given induced condition. The culture of *E. coli* NEB10B cells grew at 30 °C in M9 medium. Data are presented as mean values ± SD of n=3 biologically independent experiments with microplate photometer. The data in all right-side panels are reproduced from the data in Figure 2 and Supplementary Figure 15.

Supplementary Note 4

Regulator to neutralize dCas9 competition in other CRISPRi-based NOT-gate circuits, growth conditions, and strains

To demonstrate the effect of dCas9 competition on CRISPRi-based NOT gates in different contexts and to verify the ability of the regulated dCas9 generator to mitigate competition, we varied DNA copy number of NOT gate and competitor, *E. coli* strain, and the NOT gate input regulator molecule from the ones of the circuit in Figure 2.

First, we investigated the extent of competition and the ability of the dCas9 generator to mitigate competition when a CRISPRi-based NOT gate and a competitor sgRNA are both expressed by a plasmid with higher copy numbers. We created the plasmid pHH41 in which the origin of replication is pSC101(E93G), which was reported to be ∼84 copies/cell [S12]. As a comparison, the origin of replication of AEgPtet plasmids used in Figure 2 is pSC101, which was reported to be ∼5 copies/cell [S13]. The pHH41 plasmid encodes both the NOT gate and the competitor sgRNA cassette. When we use the BBa_J23116 promoter to transcribe the competitor sgRNA, we call this plasmid pHH41_1 (Supplementary Figure 6). When instead we use the BBa_J23100, pTrc, and BBa_J23119 promoters to transcribe the competitor sgRNA, we call the plasmids pHH41_2, pHH41_3, and pHH41_4, respectively, as listed in Supplementary Table 6. Much stronger constitutive promoters such as the pTrc and BBa_J23119 promoters [S14] were selected to reach the respective higher concentrations of the competitor sgRNA. The competitor sgRNA cassette is located in the intergenic region between the RFP expression cassette and the LuxR expression cassette. The targeting sgRNA of the NOT gate represses the strong P105 promoter of the *mRFP1* gene and is controlled by the plux promoter and transcription factor LuxR. The guide sequence of the targeting sgRNA is labeled as sgP105 (Supplementary Table 8). The pHH41 plasmids were transformed concurrently with either the pdCas9_OP or the pdCas9_CL plasmid into *E. coli* TOP10. The growth condition is at 30 °C and using glucose as the carbon source in M9610 medium. M9610 medium is buffered at pH 6 [S15].

The circuit diagrams of the constructs pOP4, pOP5, pOP6, and pOP7 are shown in Supplementary Figure 7a, where the Pc promoter is BBa_J23116, BBa_J23100, BBa_J23119, and pTrc promoter, respectively. These constructs use unregulated dCas9 generator. LuxR's effector O3-C6-HSL induces the NOT gate to repress RFP expression in the presence of different amounts of the competitor sgRNA. Dose-response curves are shown in Supplementary Figure 7b. The more the competitor sgRNA is transcribed by a stronger promoter, the more the shape of a dose-response curve deviates from the one of the curve of the pOP4 construct. Fold-changes were not computed when the concentration of HSL is higher than 30 nM because in such induced conditions, the RFP/OD values of pOP4 are approximately zero and the fold-changes become undefined. The maximal fold-change observed was up to 25-fold at 1 nM HSL when the competitor sgRNA is transcribed by the pTrc promoter. Specific growth rates at steady state were not affected across different induced conditions (Supplementary Figure 7d).

When using the regulated dCas9 generator, the constructs pCL28, pCL29, pCL30, and pCL31 should be compared to the constructs pOP4, pOP5, pOP6, and pOP7, respectively. The genetic diagram is shown in Supplementary Figure 7e. Dose-response curves of these constructs, remain practically the same even when the competitor sgRNA is transcribed by the strong pTrc promoter (Supplementary Figure 7f). The definable fold-changes remain almost equal (Supplementary Figure 7g). Comparing to the fold-changes in Supplementary Figure 7c, the regulated dCas9 generator can neutralize dCas9 competition even at higher copy numbers of the NOT gate and the competitor. Specific growth rates at steady state were barely affected across different induced conditions (Supplementary Figure 7h) and were slightly lower than the respective ones in Supplementary Figure 7d.

We next investigated whether the extent of competition and the ability of the regulated dCas9 generator to mitigate it could generalize to the use of other input regulators for the NOT gate, repressors, specifically. We thus changed the transcriptional regulator from LuxR activator to TetR repressor and the cognate promoter from plux promoter to Ptet promoter. Thus, the pHH41 plasmids were modified accordingly into what we called the pHH43 plasmids (Supplementary Figure 6). In parallel with the constructions of pOP4-pOP7 and pCL28-pCL31 constructs, we created pOP9-pOP12 and pCL33-36 constructs with the component plasmids

listed in Supplementary Table 6. They were characterized in the same growth condition as pOP4-pOP7 and pCL28-pCL31 constructs.

The circuit diagrams of the constructs pOP9, pOP10, pOP11, and pOP12 are shown in Supplementary Figure 8a, where the Pc promoter is one among BBa_J23116, BBa_J23100, BBa_J23119, and pTrc, respectively. These constructs use the unregulated dCas9 generator. TetR's effector anhydrotetracycline (aTc) induces the NOT gate to repress RFP expression in the presence of different amounts of the competitor sgRNA. Dose-response curves are shown in Supplementary Figure 8b. We could observe substantial dCas9 competition in such context, indicating that the effect of dCas9 competition is independent of the type of the regulation of the NOT gate. Fold-changes were computed as explained in Methods. The maximal fold-change up to 13-fold can be observed at 100 nM aTc when the competitor sgRNA is transcribed by the pTrc promoter. Inappreciable changes in specific growth rates at steady state were observed across different induced conditions (Supplementary Figure 8d).

When using the regulated dCas9 generator, the constructs pCL33, pCL34, pCL35, and pCL36 were built as a comparison to the constructs pOP9, pOP10, pOP11 and pOP12, respectively. The genetic diagrams are shown in Supplementary Figure 8e. From dose-response curves (Supplementary Figure 8f) and fold-changes at a given induced condition (Supplementary Figure 8g), we observed consistent results supporting that the regulated dCas9 generator can neutralize dCas9 competition in the current genetic context and growth condition. Specific growth rates at steady state were scarcely affected across different induced conditions (i.e. 0, 30, 60, 100, 300 nM aTc, Supplementary Figure 8h).

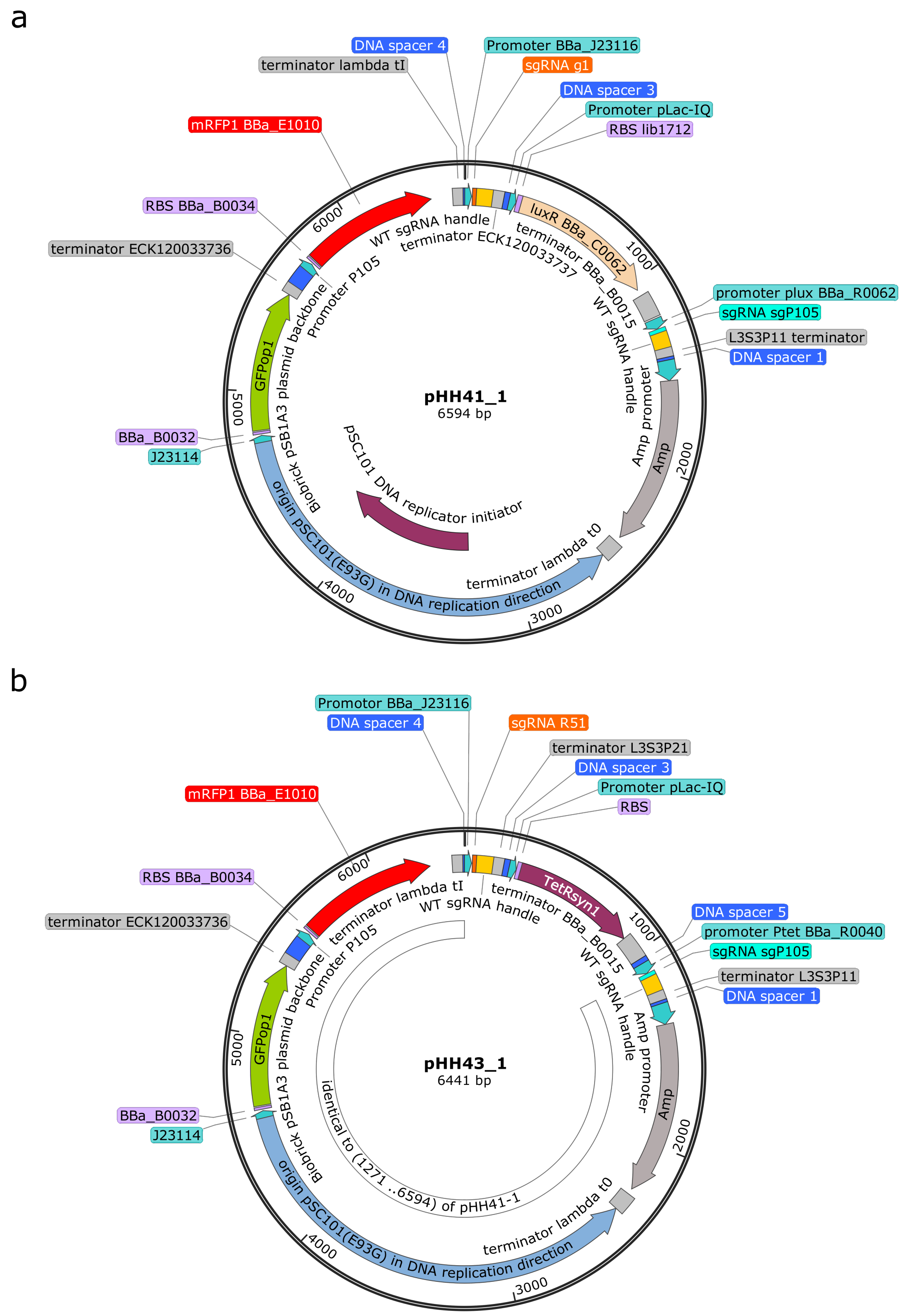

Supplementary Figure 6: **Maps of the pHH41_1 and pHH43_1 plasmids which were used in the constructs listed in Supplementary Table 6.** (a) pHH41_1 plasmid encodes a CRISPRi-based NOT gate which is regulated by transcriptional activator LuxR and the competitor sgRNA which is transcribed by BBa_J23116 promoter. The targeting sgRNA of the NOT gate and the competitor sgRNA are on map position 1271 and 40, respectively. (b) pHH43_1 plasmid encodes a CRISPRi-based NOT gate which is regulated by transcriptional repressor TetR and the competitor sgRNA which is transcribed by BBa_J23116 promoter. The targeting sgRNA of the NOT gate and the competitor sgRNA are on map position 1118 and 40, respectively. The derivation of pHH41 and pHH43 series plasmids are detailed in Supplementary Note 4. The guide sequences of sgRNAs are listed in Supplementary Table 8.

construct	plasmid 1 (Kan)	plasmid 2 (Amp)[3]	targeting sgRNA's regulator	competitor sgRNA's promoter	Figure
pOP4	pdCas9_OP	pHH41_1	LuxR	BBa_J23116	Supplementary Figure 7
pOP5		pHH41_2		BBa_J23100	
pOP6		pHH41_3		pTrc	
pOP7		pHH41_4		BBa_J23119	
pCL28	pdCas9_CL	pHH41_1		BBa_J23116	
pCL29		pHH41_2		BBa_J23100	
pCL30		pHH41_3		pTrc	
pCL31		pHH41_4		BBa_J23119	
pOP9	pdCas9_OP	pHH43_1	TetR	BBa_J23116	Supplementary Figure 8
pOP10		pHH43_2		BBa_J23100	
pOP11		pHH43_3		pTrc	
pOP12		pHH43_4		BBa_J23119	
pCL33	pdCas9_CL	pHH43_1		BBa_J23116	
pCL34		pHH43_2		BBa_J23100	
pCL35		pHH43_3		pTrc	
pCL36		pHH43_4		BBa_J23119	

Supplementary Table 6: List of the constructs used in Supplementary Figure 7 and Supplementary Figure 8. Constructs were co-transformed with the indicated plasmid 1 and plasmid 2 into *E. coli* TOP10 strain. The regulator of the targeting sgRNA and the promoter of the competitor sgRNA are listed.

[3] Maps of the pHH41_1 and pHH43_1 plasmids are shown in Supplementary Figure 6. Variants of pHH41 and pHH43 plasmids are only different in the promoter of the competitor sgRNA. The usage of these constructs is detailed in Supplementary Note 4.

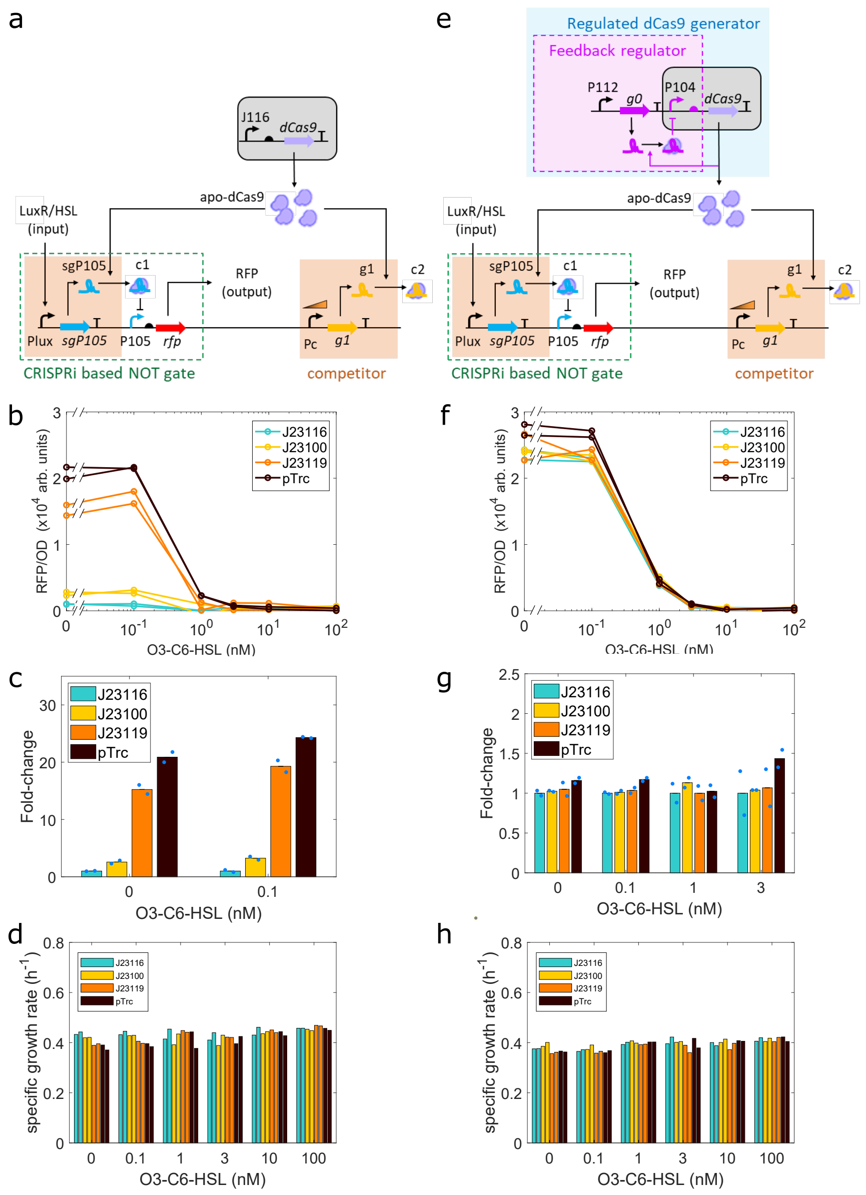

Supplementary Figure 7: **Neutralization of dCas9 competition in the high-copy number CRISPRi-based NOT gate.** The panels (a-d) and (e-h) exhibit the results when using the unregulated and regulated dCas9 generator, respectively. (a) Genetic circuit of the CRISPRi-based NOT gate and the competitor sgRNA is encoded in a plasmid using pSC101(E93G) origin (∼84 copies). The Pc promoter of the competitor module is BBa_J23116, BBa_J23100, BBa_J23119, or pTrc promoter to tune the level of the competitor sgRNA. Apo-dCas9 proteins are expressed from the unregulated dCas9 generator in a plasmid using p15A origin. (b) Comparison of dose-response curves in the presence of different amounts of the competitor sgRNA transcribed by the indicated promoter. (c) Fold-changes at a given HSL induction were computed as described in Methods section Quantification of competition effects, by dividing the RFP/OD value of a construct by the one of the construct which uses the BBa_J23116 promoter to transcribe the competitor sgRNA. (d) Specific growth rates of each construct at a given induced condition. (e) Genetic circuit of the CRISPRi-based NOT gate and the competitor is the same as in (a). Apo-dCas9 proteins are expressed from the regulated dCas9 generator in a plasmid using p15A origin. (f) Comparison of dose-response curves in the presence of different amounts of the competitor sgRNA transcribed by the indicated promoter. (g) Fold-changes were computed in the same way as in (c). (h) Specific growth rates of each construct at a given induced condition. The culture of *E. coli* TOP10 cells grew at 30 °C in M9610 medium. Data are presented for n=2 biologically independent experiments with microplate photometer.

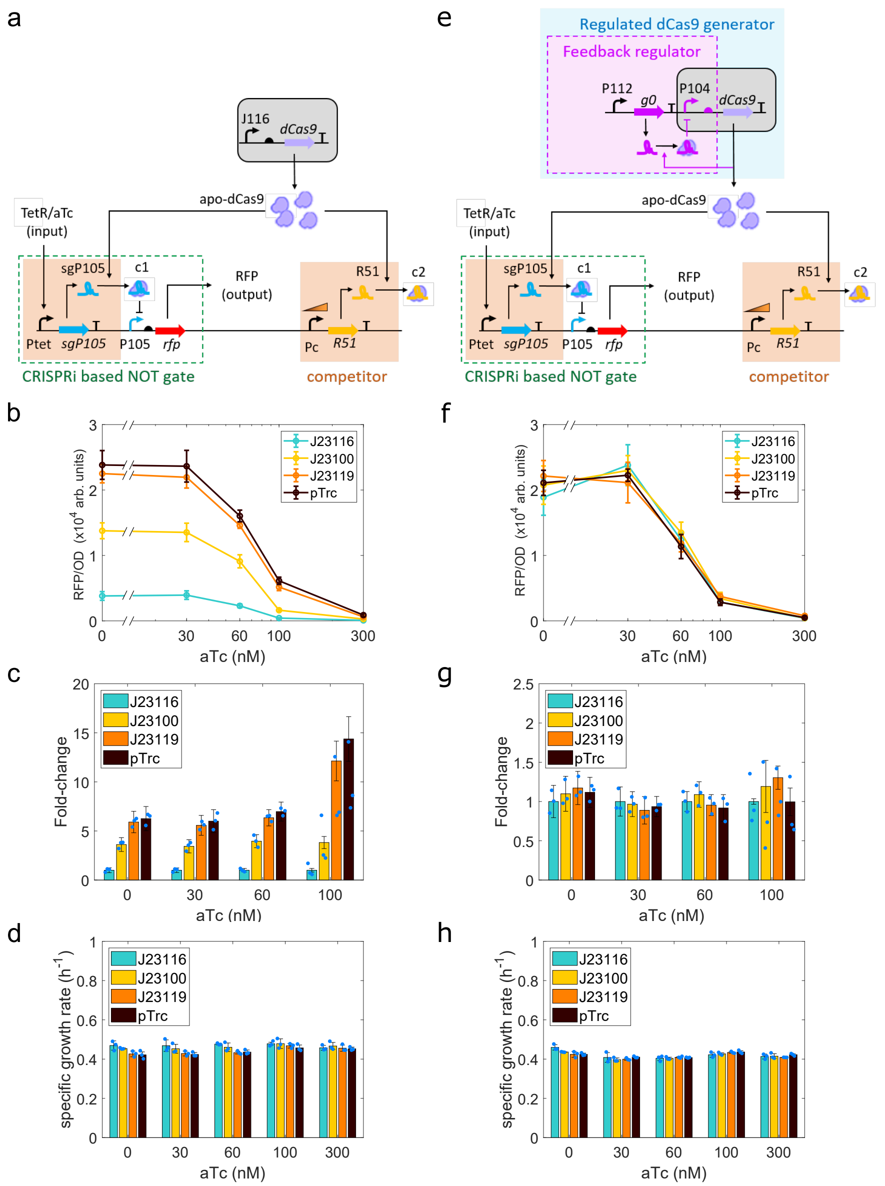

Supplementary Figure 8: **Neutralization of dCas9 competition in the high copy number CRISPRi-based NOT gate with input regulator TetR and its effector aTc.** The panels (a-d) and (e-h) exhibit the results when using the unregulated and regulated dCas9 generator, respectively. (a) Genetic circuit of the CRISPRi-based NOT gate and the competitor sgRNA is encoded in a plasmid using pSC101(E93G) origin. The Pc promoter of the competitor module is the BBa_J23116, BBa_J23100, BBa_J23119, or pTrc promoter to tune the level of the competitor sgRNA. Apo-dCas9 proteins are expressed from the unregulated dCas9 generator in a plasmid using p15A origin. (b) Comparison of dose-response curves in the presence of different concentrations of the competitor sgRNA transcribed by the indicated promoter. (c) Fold-changes at a given aTc induction were computed as described in Methods section Quantification of competition effects, by dividing the RFP/OD value of a construct by the one of the construct which uses the BBa_J23116 promoter to transcribe the competitor sgRNA. (d) Specific growth rates of each construct at a given induced condition. (e) Genetic circuit of the CRISPRi-based NOT gate and the competitor is the same as in (a). Apo-dCas9 proteins are expressed from the regulated dCas9 generator in a plasmid using p15A origin. (f) Comparison of dose-response curves in the presence of different concentrations of the competitor sgRNA transcribed by the indicated promoter. (g) Similarly, fold-changes were computed in the same way as in (c). (h) Specific growth rates of each construct at a given induced condition. The culture of *E. coli* TOP10 cells grew at 30 °C in M9610 medium. Data are presented as mean values ± SD of n=3 biologically independent experiments with microplate photometer.

Supplementary Note 5

DNA sequences

DNA sequences of the plasmids listed in Supplementary Table 7 can be found in Supplementary Data. The guide sequences of all sgRNAs are listed in Supplementary Table 8. All designs of sgRNA sequences were aided with the CRISPR Guide RNA Design Tool of Benchling (Benchling.com).

plasmid[4]	antibiotic resistence[5]	replication origin	copy number per cell[6]
pdCas9_OP	Kan	p15A	20
pdCas9_CL	Kan	p15A	20
pdCas9_CL2	Kan	p15A	20
pdCas9_CL7	Kan	p15A	20
I13521-target	Amp	pUC19-derived pMB1	100+[7]
AEgPtet_No_competitor	Cm	pSC101	5
AEgPtetJ116gplac	Cm	pSC101	5
AEgPtetJ100gplac	Cm	pSC101	5
pHH50-I	Amp	pSC101(E93G)	84
pHH50-IV	Amp	pSC101(E93G)	84
pAUX_OL	Amp	oriR101 w/repA101ts	n.a.
pHH41_NOg2	Amp	pSC101(E93G)	84
pHH41_1	Amp	pSC101(E93G)	84
pHH41_2	Amp	pSC101(E93G)	84
pHH41_3	Amp	pSC101(E93G)	84
pHH41_4	Amp	pSC101(E93G)	84
pHH43_1	Amp	pSC101(E93G)	84
pHH43_2	Amp	pSC101(E93G)	84
pHH43_3	Amp	pSC101(E93G)	84
pHH43_4	Amp	pSC101(E93G)	84

Supplementary Table 7: List of plasmids used in this work.

sgRNA	20-nt guide sequence	intended target[8]	encoded plasmid	functional role[9]
g0	agtattgactattaatcatc	P104 promoter	pdCas9_CL	repress dCas9 expression
g1	tgtcaatctctatcactgat	pTet promoter (BBa_R0040)	AEgPtet series, pHH50-I, pHH50-IV	repress RFP expression
g2	ataacaattgacattgtgag	plac promoter (BBa_R0011)	AEgPtet series, pHH41 series, pHH50-I	sequester apo-dCas9
g3	gaatctattatatcgccgca	P075 promoter	pHH50-I, pHH50-IV	NOT gate cascade
sg3	aacgtagcatgtagatccga	none	pHH48-I	sequester apo-dCas9
sg4	ggatatcgttatgctactat	none	pHH48-I, pHH55-I	sequester apo-dCas9
R51	ggtaaaatagtcaacacgca	lambda cI promoter (BBa_R0051)	pHH43 series	sequester apo-dCas9
sgP105	gaaaaattttcctgatgtca	P105 promoter	pHH41 series, pHH43 series	repress RFP expression

Supplementary Table 8: List of all sgRNAs used in this work.

4 The DNA sequence of the strong ribosome binding site RBS1 used in the pdCas9_CL is as CTCTAGACGAGAG-GAAACGCGGTTTTAATATAAGGAGGTTATTA. The predicted maximal translation initiation rate (TIR) is 1384771 designed by the RBS calculator 2.0 [S11].

5 Kan, Amp, and Cm stand for kanamycin, ampicillin, and chloramphenicol, respectively.

6 Data are adopted from [S12]. n.a., not available.

7 The copy number of pUC19-derived pMB1 origin was reported as 500-700 from [S16] or 100-300 from the iGEM information on pSB1A2 plasmid from the Standard Registry of Biological parts.

8 The P075, P104, and P105 promoters are adopted from Ec-TTL-P075, P104, and P105 promoters [S14], respectively. Other promoters with a BBa_ number are adopted from iGEM registry.

9 For the competitor sgRNAs, the intended target sequence is absent in the indicated plasmids and host cell strain.

Supplementary Note 6

Genetic constructs in the main text

A genetic construct (Figure 2b and 2c), in which the CRISPRi-based NOT gate competes with a different amount of the competitor sgRNA g2 for apo-dCas9 proteins produced by the unregulated dCas9 generator, was implemented with the constructs pOP94, pOP91, and pOP92 listed in Supplementary Table 9. Specifically, each construct is composed of the indicated two plasmids. The pdCas9_OP plasmid encodes the unregulated dCas9 generator and is the plasmid j116-dcas9-3k3 (BBa_J107202) from [S17]. The NOT gate is implemented as a CRISPRi-based module (CBM). Both the NOT gate and its CRISPRi target cassette locate in the second plasmid such as the pHH41_NOg2, pHH41_2, or pHH41_3 plasmid (See Supplementary Note 4). The pHH41_NOg2 plasmid is lack of the competitor sgRNA g2 cassette. The pHH41 plasmids use the replication origin pSC101(E93G) [S12] as a high copy (HC) number plasmid (~ 84). When the respective pHH41 plasmid is co-transformed with the pdCas9_CL plasmid, the constructs pCL112, pCL109, and pCL110 as the list in Supplementary Table 9 were built. The pdCas9_CL plasmid encodes the regulated dCas9 generator and is constructed by Gibson assembly as shown in the Supplementary Table 12.

Another genetic construct (Figure 2d and 2e), in which the CRISPRi-based NOT gate competes for apo-dCas9 proteins produced by the unregulated dCas9 generator, was implemented with the constructs pOP69, pOP70, and pOP71 listed in Supplementary Table 10. Specifically, each construct is composed of the pdCas9_OP plasmid and the indicated two plasmids. The NOT gate is implemented in an AEgPtet plasmid and its target cassette is in the I13521-target plasmid. The AEgPtet plasmids use the replication origin pSC101 [S12] as a low copy (LC) number plasmid (~ 5). The competitor sgRNA g2 cassette is absent in the AEgPtet_No_competitor plasmid of the construct pOP69, but is present in the AEgPtet_116gPlac and AEgPtet_100gPlac plasmids of the constructs pOP70 and pOP71, respectively. The AEgPtet_116gPlac and AEgPtet_100gPlac plasmids use BBa_J23116 and BBa_J23100 promoters, respectively, to transcribe the competitor sgRNA g2. Similarly, the genetic circuit in Figure 2e was implemented with the constructs pCL87, pCL88, and pCL89, respectively. The construction of pCL112, pCL109, pCL110, pCL87, pCL88, and pCL89 requires the usage of pAUX_OL plasmid which is detailed in Supplementary Note 2.

construct	plasmid 1 (Kan)	plasmid 2 (Amp)	sgRNA g2's promoter
pOP94	pdCas9_OP	pHH41_NOg2	cassette absent
pOP91		pHH41_2	BBa_J23100
pOP92		pHH41_3	pTrc
pCL112	pdCas9_CL	pHH41_NOg2	cassette absent
pCL109		pHH41_2	BBa_J23100
pCL110		pHH41_3	pTrc

Supplementary Table 9: List of the constructs used in Figure 2b and 2c. Each construct results from concurrent transformation of the indicated two plasmids into *E. coli* NEB10B strain. The plasmids 1 and 2 have antibiotic resistance kanamycin and ampicillin, respectively, and the origin of replication as p15A and pSC101(E93G) [S12], respectively.

[10] All plasmids of the plasmid groups 2 and 3 are from [S17].

[11] The BBa_J23116 and BBa_J23100 promoters are from the iGEM Registry of Standard Biological Parts. Maps of the plasmids 1, 2, and 3 are shown in Supplementary Figure 9 and Supplementary Figure 10.

construct	plasmid 1 (Kan)	plasmid 2 (Amp)	plasmid 3 (Cm)[10]	sgRNA g2's promoter[11]
pOP69	pdCas9_OP	I13521-target	AEgPtet_No_competitor	cassette absent
pOP70			AEgPtet_116gPlac	BBa_J23116
pOP71			AEgPtet_100gPlac	BBa_J23100
pCL87	pdCas9_CL		AEgPtet_No_competitor	cassette absent
pCL88			AEgPtet_116gPlac	BBa_J23116
pCL89			AEgPtet_100gPlac	BBa_J23100

Supplementary Table 10: List of the constructs used in Figure 2d and 2e. Each construct was obtained by co-transformation of the three indicated plasmids into *E. coli* NEB10B strain. The component plasmids 1, 2, and 3 have antibiotic resistance kanamycin, ampicillin, and chloramphenicol, respectively, and have the origin of replication as p15A [S12], pUC19-derived pMB1 [S16], and pSC101 [S12], respectively.

The genetic cirucit (Figure 3b), in which the CRISPRi-based NOT gate cascade operates with and without the competitor sgRNA g2 for apo-dCas9 proteins produced by the unregulated dCas9 generator, was built as the constructs pOP64 and pOP65, respectively, as listed in Supplementary Table 11. Specifically, each construct is composed of the two indicated plasmids. pHH50-IV encodes the NOT gate cascade and does not encode the competitor sgRNA. pHH50-I is derived from pHH50-IV by introducing the competitor sgRNA g2 in downstream of the P108 promoter of pHH50-IV. The P108 promoter is adopted from the Ec-TTL-P108 promoter [S14]. Similarly, the genetic circuits using the regulated dCas9 generator with or without the competitor sgRNA were built as the constructs pCL82 and pCL83, respectively. The construction of pCL82 and pCL83 requires the usage of pAUX_OL plasmid and is detailed in Supplementary Note 2. Gibson assembly and maps of the pHH50-I and pHH50-IV plasmids are shown in the Supplementary Table 12 and in the Supplementary Figure 11, respectively.

construct	plasmid 1 (Kan)	plasmid 2 (Amp)	competitor sgRNA g2[12]
pOP64	pdCas9_OP	pHH50-I	encoded
pOP65		pHH50-IV	not encoded
pCL82	pdCas9_CL	pHH50-I	encoded
pCL83		pHH50-IV	not encoded

Supplementary Table 11: List of the constructs used in Figure 3. Each construct results from concurrent transformation of the indicated two plasmids into *E. coli* NEB10B strain. The plasmids 1 and 2 have antibiotic resistance kanamycin and ampicillin, respectively, and the origin of replication as p15A and pSC101(E93G) [S12], respectively.

[12] The pHH50-I plasmid encodes the competitor sgRNA g2 cassette (i.e. promoter, sgRNA, and terminator). The pHH50-IV plasmid keeps the P108 promoter and the synthetic terminator 4 of the cassette but sgRNA g2 is absent.

Plasmid	Fragment	Forward	Reverse	DNA Template	Size (bp)
pdCas9_CL	1	P264	P312	P112_sgRNA_P104_dCas9_B34	2143
	2	P311	AH1prR	P112_sgRNA_P104_dCas9_B34	2186
	3	AH2prF	P263	P112_sgRNA_P104_dCas9_B34	1318
	4	P262	GFP-VR	P112_sgRNA_P104_dCas9_B34	1644
pHH50-I	1	Load_F2	P662	pHH48-I #2	2424
	2	P663	P664	2ndStage_RFP	120
	3	P665	P730	2ndStage_RFP	534
	4	P731	P667	pHH48-I #2	1090
	5	P668	P367	AEgPtetYgpComp-double-knob	1129
	6	P669	Load_R2	pHH48-I #2	1855
pHH50-IV	1	Load_F2	P662	pHH48-I #2	2424
	2	P663	P664	2ndStage_RFP	120
	3	P665	P730	2ndStage_RFP	534
	4	P731	P667	pHH48-I #2	1090
	5	P668	P367	AEgPtetYgpComp-double-knob	1129
	6	P669	P613	pHH48-I #2	351
	7	P614	Load_R2	pHH48-I #2	1442

Supplementary Table 12: List of plasmids which were assembled by Gibson assembly and used in Supplementary Table 10 and Supplementary Table 11 . Each fragment was prepared by the indicated forward and reverse primers and the DNA template.

Promoter	Use	Strength	Plasmid	Reference
BBa_J23116	dCas9 expression in unregulated systems<br>g2 weak expression in NOT gate systems	1	pdCas9_OP<br>AEgPtet_116gPlac	[S14]
BBa_J23100	g2 strong expression in NOT gate systems	20	AEgPtet_100gPlac	[S14]
P104	dCas9 expression in regulated systems	130	pdCas9_R	[S14]
P112	g0 expression in regulated systems	258	pdCas9_R	[S14]
P108	g2 expresison in Cascade systems	173	pHH50-IV	[S14]
Ptet	RFP expression in NOT gate system<br>g3 expression in Cascades system	34	I13521-target<br>pHH50-I,pHH50-IV	[S14]
P075	RFP expression in Cascade systems	48	pHH50-I, pHH50-IV	[S14]
Plux[13]	g1 expression in all the reported systems	97	AEgPtet_No_competitor<br>AEgPtet_116gPlac<br>AEgPtet_100gPlac<br>pHH50-I, pHH50-IV	[S14, S17]

Supplementary Table 13: Promoters used in the main text with relative strength, as evaluated in [S14] and [S17], normalized to BBa_J23116.

[13] This Plux carries a deletion of the last 3 nucleotides (AAA) to set the transcription start site at +1; the deletion leads to a reduction of protein synthesis rate of ∼3.7 times, as reported in [S17]

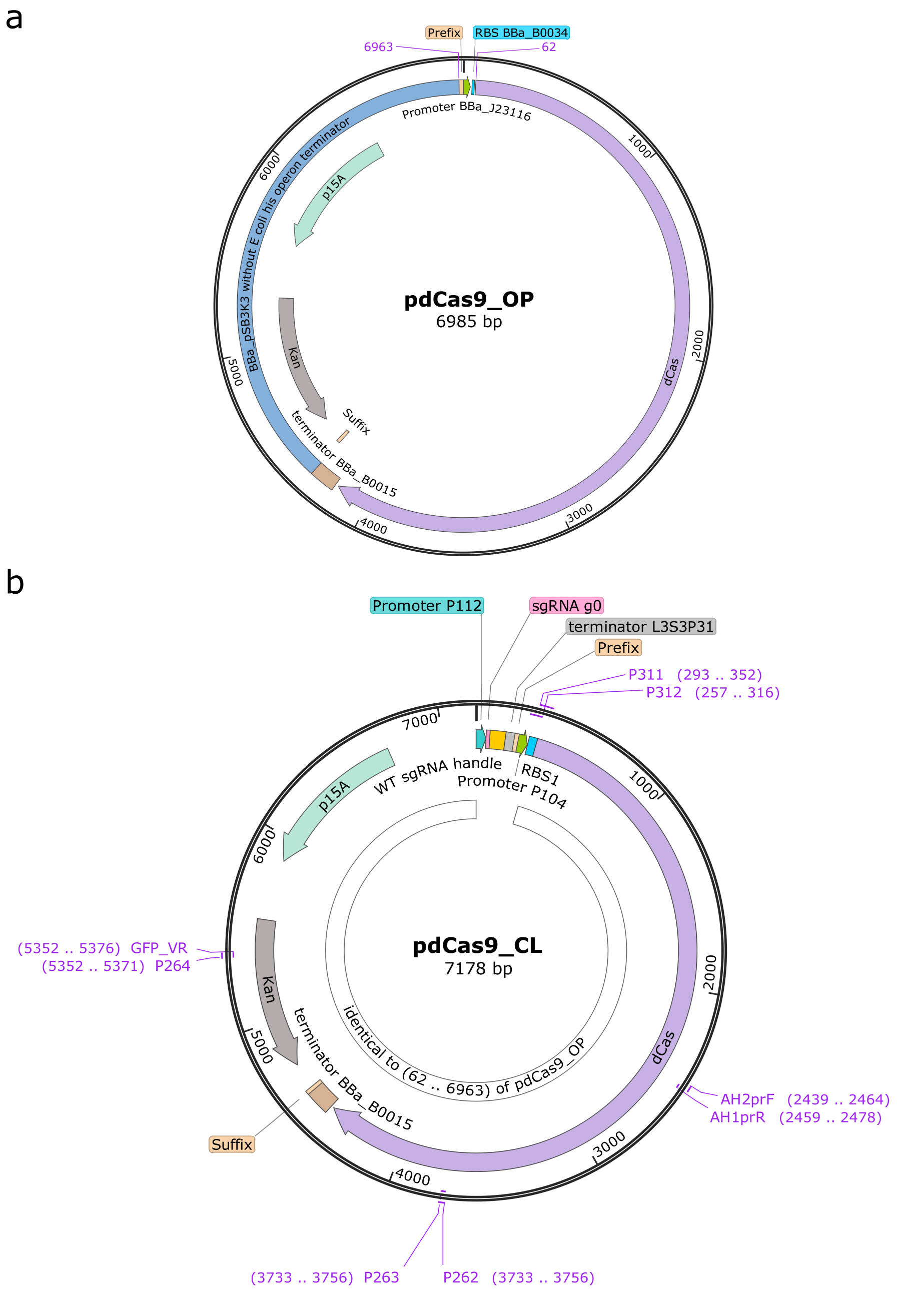

Supplementary Figure 9: **Maps of the pdCas9_OP and pdCas9_CL plasmids used in the constructs listed in Supplementary Table 10, Supplementary Table 11, and Supplementary Table 6.** (a) The pdCas9_OP plasmid uses the promoter BBa_J23116 and the RBS BBa_B0034 to constitutively express dCas9 protein. The annotated map section from 62 to 6963 is identical to the pdCas9_CL plasmid. (b) The pdCas9_CL plasmid uses the regulator as shown in Figure 1b to control the expression of dCas9 protein. Specifically, the regulator comprises the strong promoter P104 and the strong RBS *RBS*1 to express dCas9 protein and uses the strong promoter P112 to constitutively transcribe the sgRNA g0, which targets the promoter P104 of the *dCas9* gene to render the regulation. The P104 and P112 promoters are adopted from the Ec-TTL-P104 and Ec-TTL-P112 promoters, respectively [S14]. The sgRNA g0 is composed of the 20-nt sgRNA g0 sequence, the wild-type (WT) sgRNA handle [S18], and the terminator L3S3P31 [S19]. Furthermore, the 20-nt sgRNA g0 sequence was designed with the CRISPR Guide RNA Design Tool of Benchling (Benchling.com). The cloning primers listed in Supplementary Table 12 are annotated as a purple text with the respective map position.

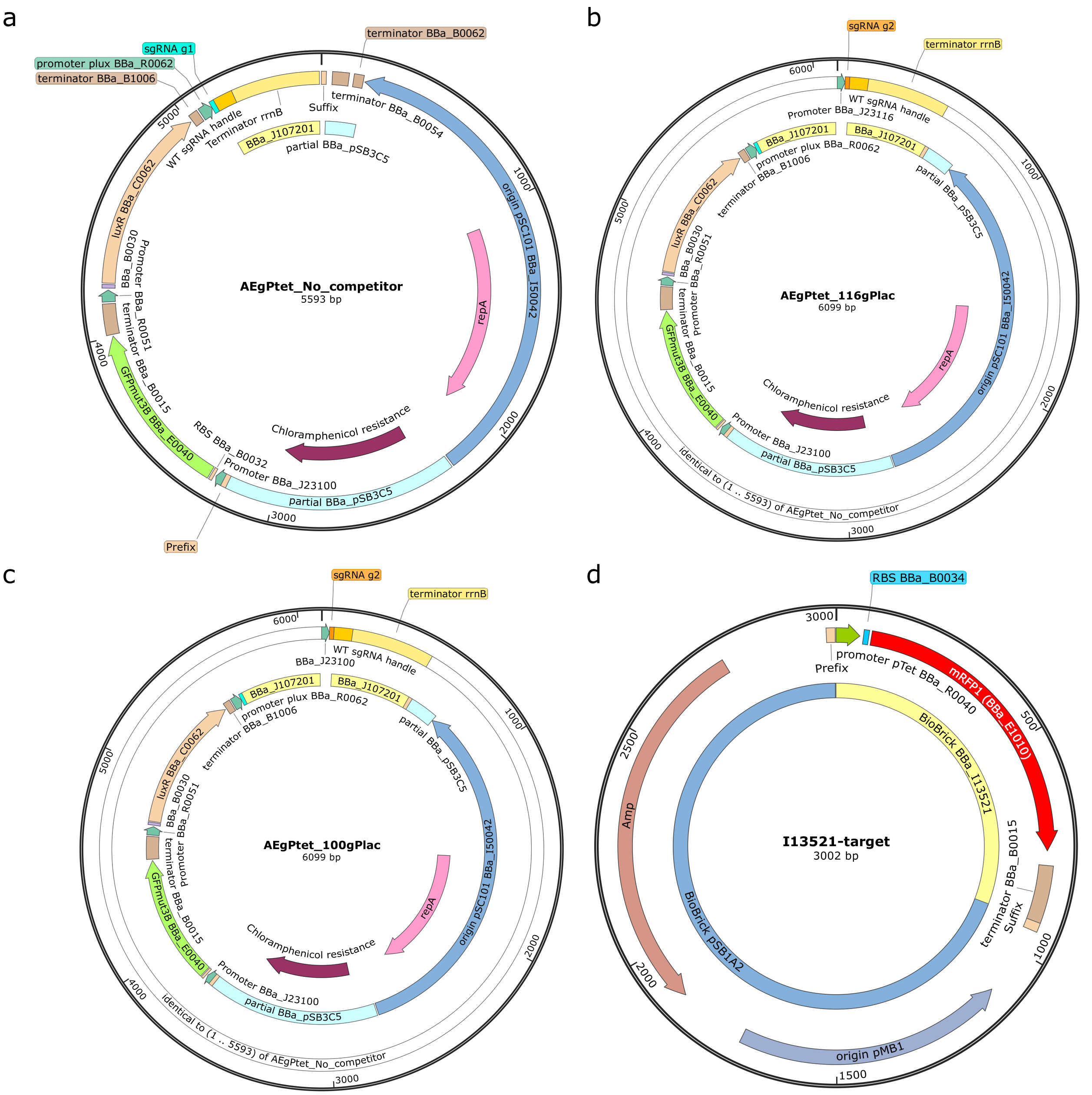

Supplementary Figure 10: **Maps of the AEgPtet and I13521-target plasmids which were used in the constructs listed in Supplementary Table 10.** (a-c) In the AEgPtet plasmid, specifically, the NOT gate uses transcriptional activator LuxR and its effector HSL (i.e. LuxR/HSL) as the input and red fluorescent protein RFP as the output. LuxR/HSL activates the Plux promoter to transcribe the sgRNA g1 from the AEgPtet plasmid. The dCas9-sgRNA g1 complex represses the pTet promoter of the *mRFP1* gene in the I13521-target plasmid. AEgPtet_No_competitor does not encode the competitor sgRNA cassette. AEgPtet_116gPlac and AEgPtet_100gPlac plasmids use the BBa_J23116 and the BBa_J23100 promoters to constitutively transcribe the sgRNA g2, respectively. (d) The I13521-target plasmid uses the promoter pTet to constitutively express red fluorescence protein (mRFP1). The 20-nt guide sequences of the sgRNAs g1 and g2 were designed with the CRISPR Guide RNA Design Tool of Benchling (Benchling.com) to target the pTet and BBa_R0011 promoters, respectively, without predicted off-targets in the genome of *E. coli* K-12 strain. Note that the BBa_R0011 promoter is not used in any plasmid of this work. The sgRNA g1 and g2 have the common BBa_J107201 BioBrick part which includes the WT sgRNA handle and the terminator rrnB.

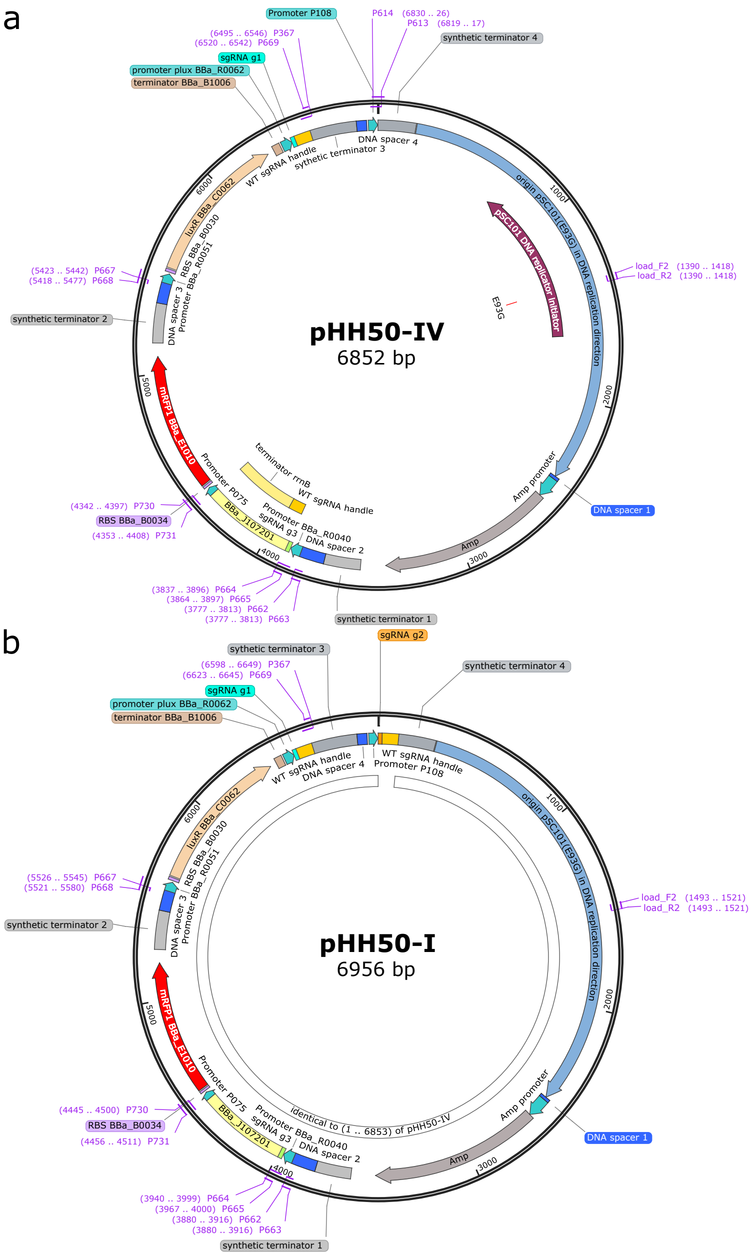

Supplementary Figure 11: **Maps of the pHH50-IV and pHH50-I plasmids which were used in the constructs listed in Supplementary Table 11.** (a) The CRISPRi-based NOT gate cascade in Figure 3b is implemented in pHH50-IV and uses transcriptional activator LuxR and its effector HSL (i.e. LuxR/HSL) as the input and red fluorescent protein RFP as the output. Specifically, LuxR/HSL activates the plux promoter (BBa_R0062 on map position 6365) to transcribe the sgRNA g1 (on map position 6417) to target the pTet promoter. The first-stage NOT gate uses the pTet promoter (BBa_R0040 on map position 3816) to transcribe the sgRNA g3 (on map position 3871) to target the P075 promoter. The second-stage NOT gate uses the P075 promoter (on map position 4342) to express *mRFP1* gene as the output of the cascade. The P108 promoter is located immediately upstream of the synthetic terminator 4. No competitor sgRNA is encoded in this plasmid. The P075 and P108 promoters are adopted from the Ec-TTL-P075 and Ec-TTL-P108 promoters [S14]. (b) The pHH50-I plasmid encodes the 20-nt guide sequence of the competitor sgRNA g2 (on map position 1) and the WT sgRNA handle (on map position 21) in downstream of the P108 promoter (on map position 6910). The sgRNA g3 was designed in the same way as the sgRNAs g1 and g2 but only differs in the 20-nt guide sequence. The cloning primers listed in Supplementary Table 12 are annotated with purple text with the respective map position.

Flow cytometry data for high copy CIRSPRi-based NOT gate

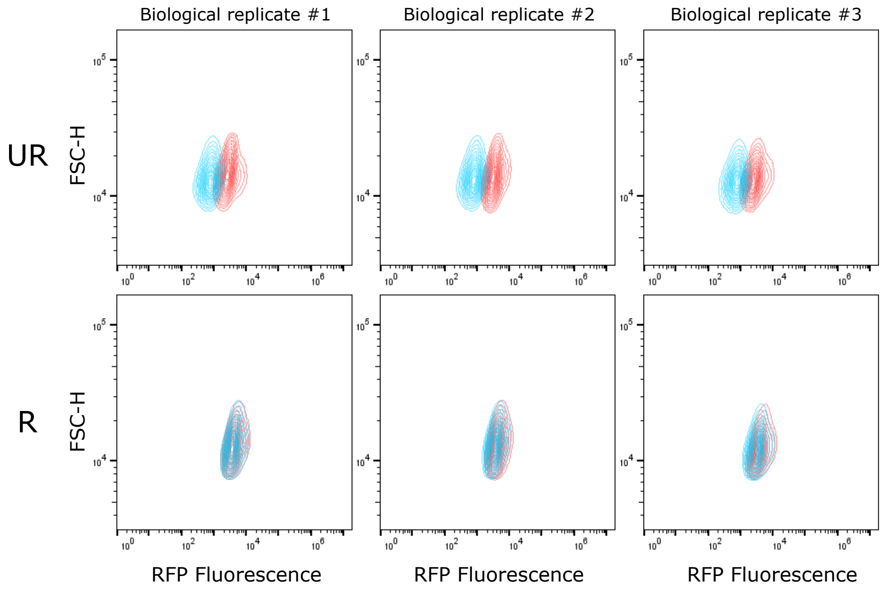

Supplementary Figure 12: **Flow cytometry data for the NOT gate circuit.** Flow cytometry data for the NOT gate circuit without O3-C6-HSL induction in Figure 2b and 2c. The populations of the constructs using 'low competitor' and 'higher competitor' modules are represented by the contours in blue and red colors, respectively. Three biological replicates are measured by Accuri C6 flow cytometer (Becton Dickinson, Special Order 2B2LYG RUO System, 656035) to generate contour plots of FSC-H (forward scattering channel-Height) versus RFP fluorescence. The detection threshold was set as 7000 on FSC-H channel. The singlet events are at least 70000 counts and analyzed by the FlowJo v10 (FlowJo, LLC).

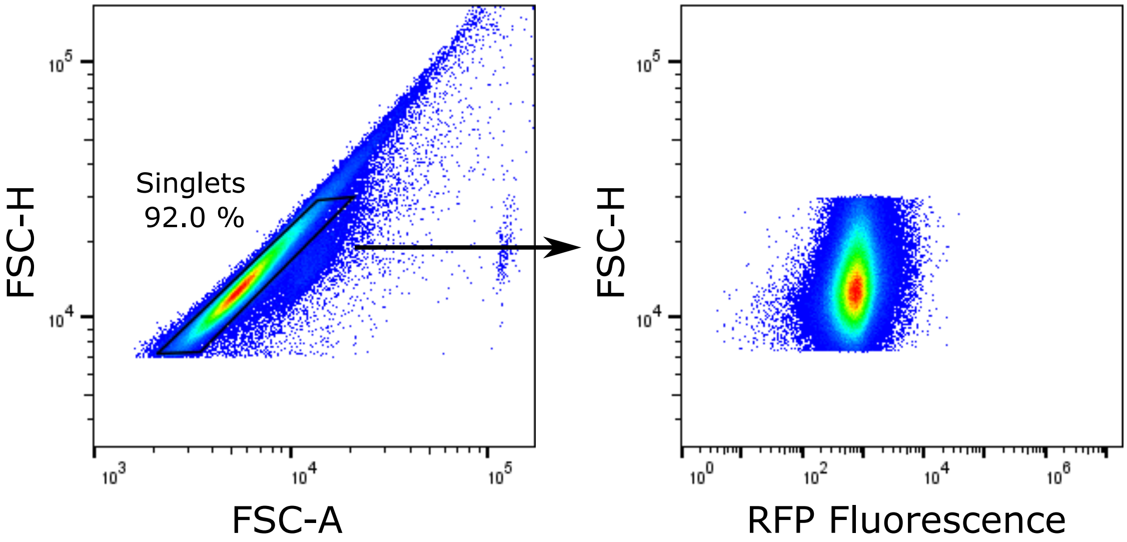

Supplementary Figure 13: **Gating strategy.** The singlet events are first gated in a FSC-H versus FSC-A plot. The pseudocolor plots of FSC-H versus RFP fluorescence of the singlet events are analyzed.

Supplementary Note 7

Specific growth rates of the experiments in the main text

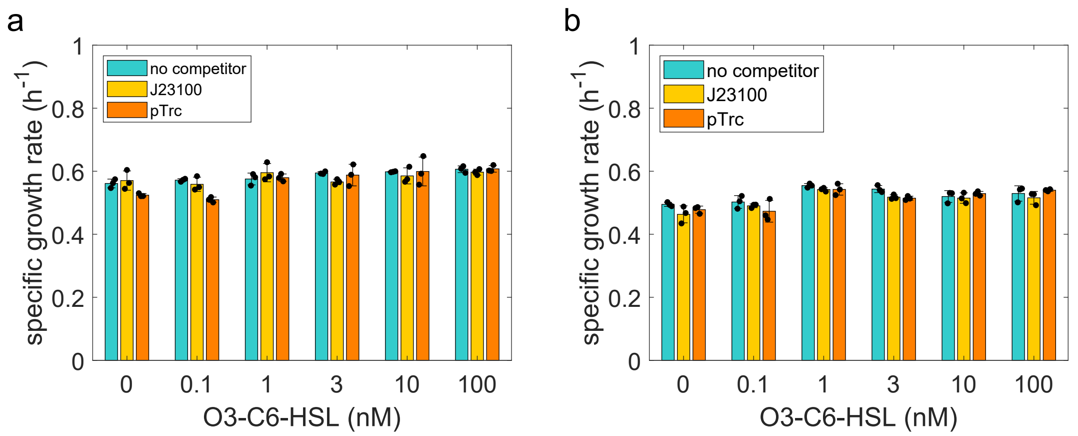

Supplementary Figure 14: **Specific growth rates observed in the experiments of the NOT gate circuit in Figure 2b and 2c.** (a) The rates at steady state were observed in the data of Figure 2b. (b) The rates at steady state were observed in the data of Figure 2c. Data are presented as mean values ± SD of n=3 biologically independent experiments.

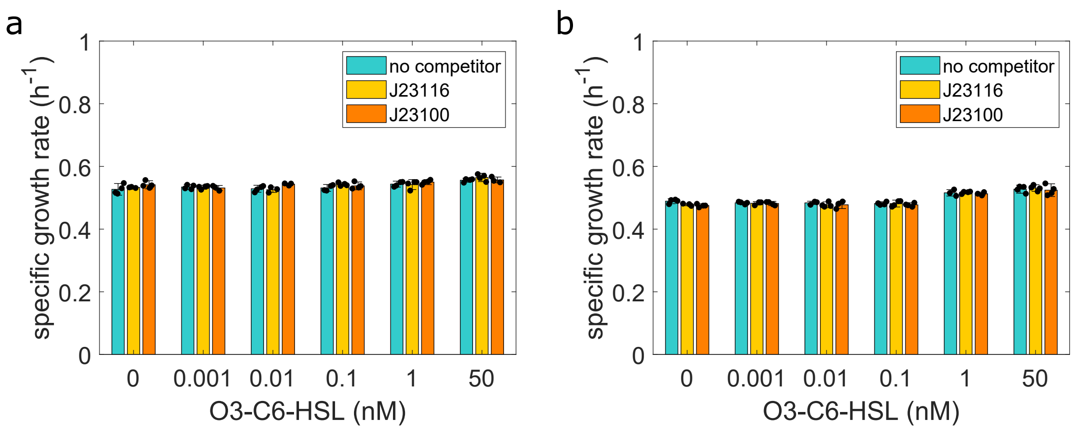

Supplementary Figure 15: **Specific growth rates observed in the experiments of the NOT gate circuit in Figure 2d and 2e.** (a) The rates at steady state were observed in the data of Figure 2d. (b) The rates at steady state were observed in the data of Figure 2e. Data are presented as mean values ± SD of n=4 biologically independent experiments.

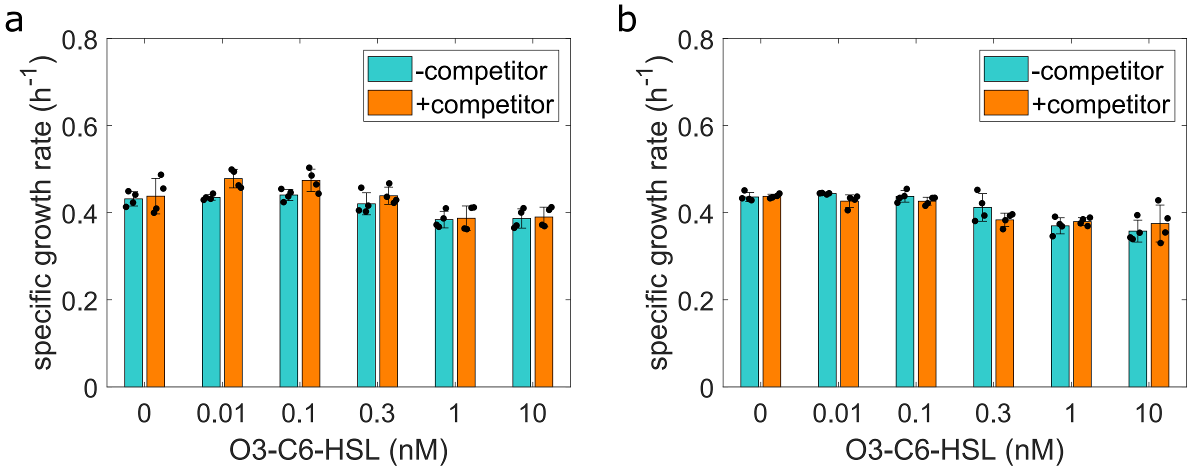

Supplementary Figure 16: **Specific growth rate observed in the experiments of the two-stage NOT gate cascade in Figure 3b.** (a) The rates at steady state were observed in the data of Figure 3c. (b) The rates at steady state were observed in the data of Figure 3d. Data are presented as mean values ± SD of n=4 biologically independent experiments.

Supplementary Note 8

Repression thresholds of the NOT gates with unregulated dCas9 generator

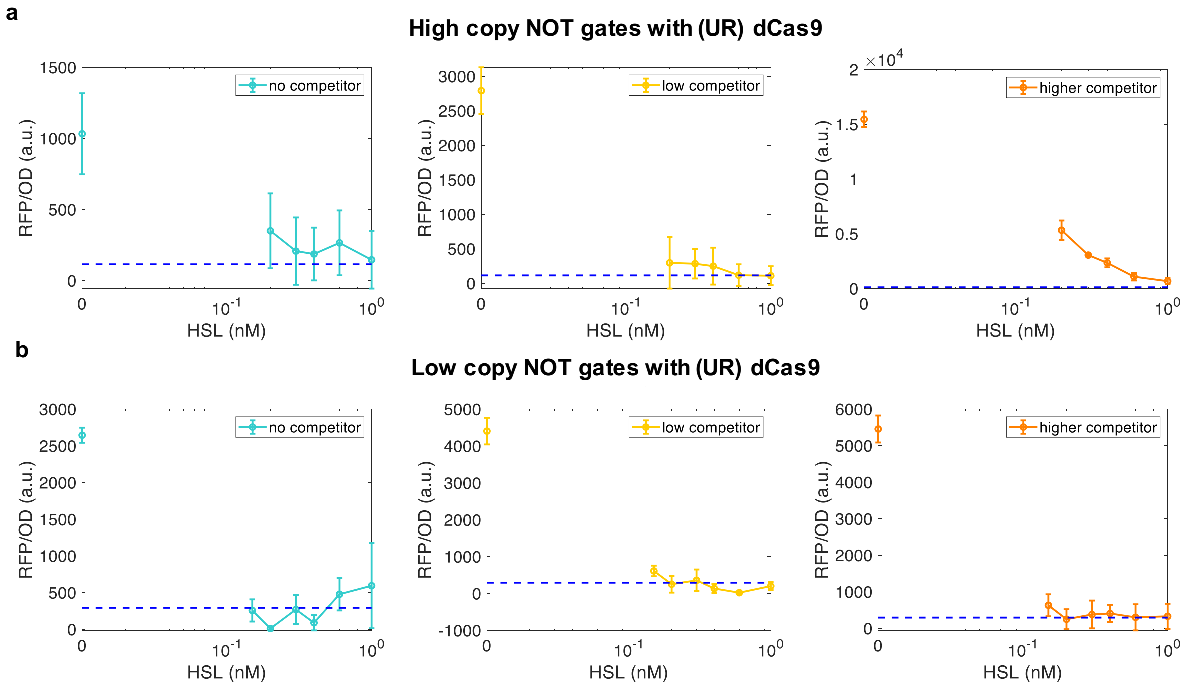

Supplementary Figure 17: **Data for HSL levels within the interval [0.1nM, 1nM], that is, around the I/O response knees of Figure 2b and 2d.** The dashed horizontal line represents 10% of the RFP/OD value corresponding to 0nM HSL for the 'no competitor' condition. (a) The RFP/OD at steady state for the high copy NOT gate, corresponding to the data of Figure 2b. (b) The RFP/OD at steady state for the low copy NOT gate, corresponding to the data of Figure 2d. Data are presented as mean values ± SD of n=3 biologically independent experiments. Negative replicates arising from blanking RFP measurements with values close to the minimum machine detection boundaries were set to zero.

We define the repression threshold (RT) as the minimum HSL level required to obtain an RFP/OD output that is at or below 10% of the RFP/OD value obtained for HSL = 0nM in the 'no competitor' condition. The RT in the high-copy NOT gate has values ∼0.2nM for no competitor and low competitor, and greater than 1nM for high competitor. For the low copy NOT gate, the RT is ∼0.2nM for no competitor, and ∼0.3nM for low and high competitor. Hence, the RT increases with the addition of competitor sgRNA.

Supplementary Note 9

Multiple batch data of the NOT gates in the main text

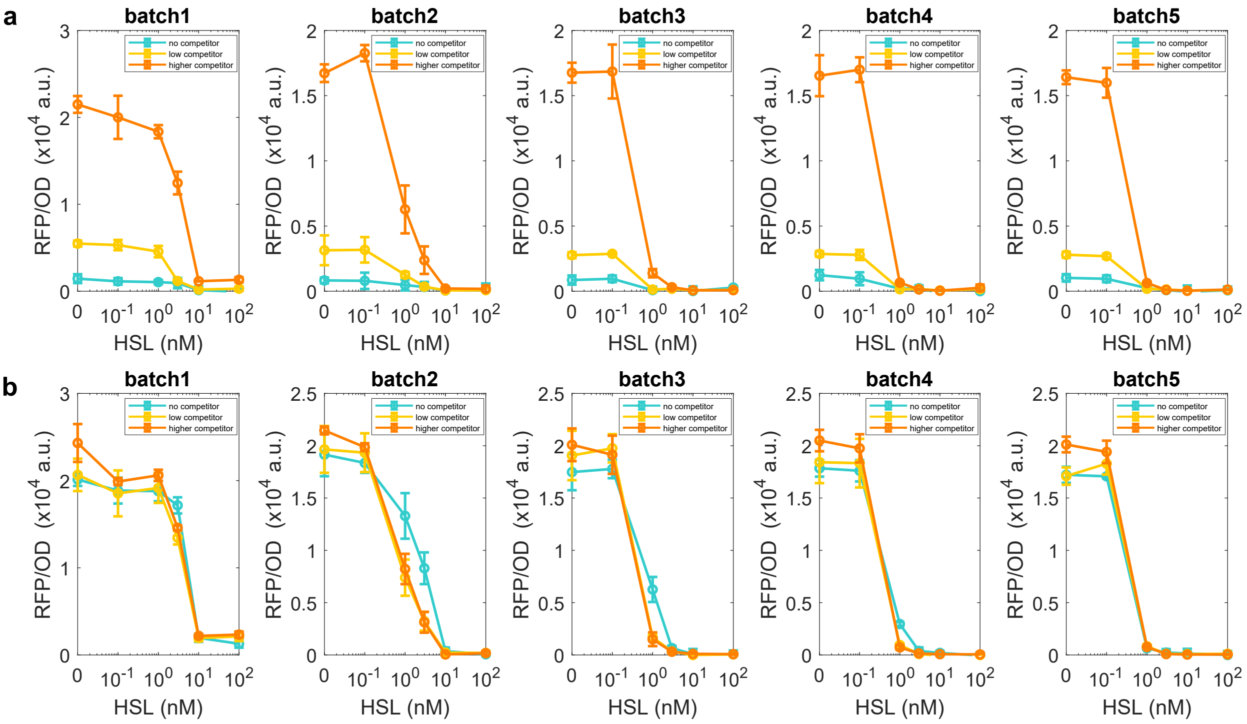

Supplementary Figure 18: **RFP/OD data for high copy unregulated (UR - panel a) and regulated (R - panel b) NOT gate in Figure 2b and 2c.** Data are presented as mean values ± SD of n=3 biologically independent experiments with microplate photometer.

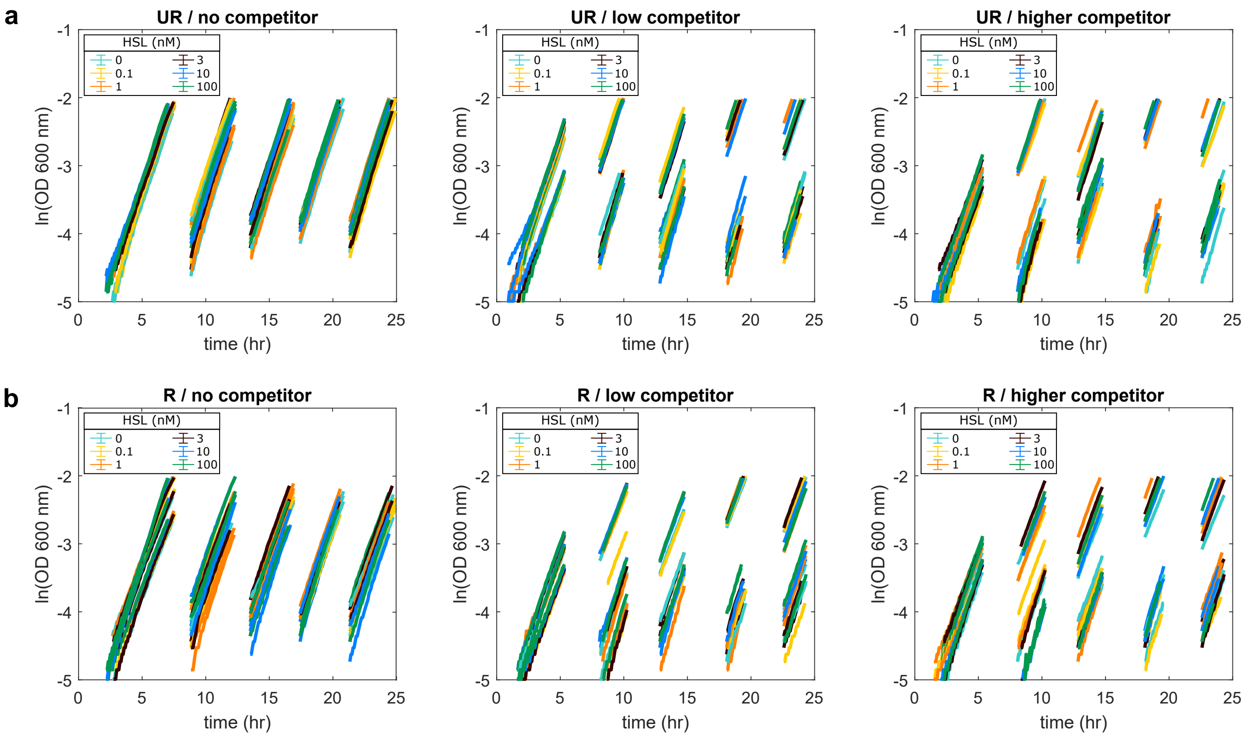

Supplementary Figure 19: **Exponential growth data for high copy unregulated (UR - panel a) and regulated (R - panel b) NOT gate in Figure 2b and 2c.** Each trace represents the growth curve of an independent experiment. Three biological replicates are shown in the same color as depicted in the figure legends. Linearity of each growth curve confirms the exponential growth. To analyze RFP/OD values, we choose the data point with an OD value most close to 0.0598 for each experimental condition.

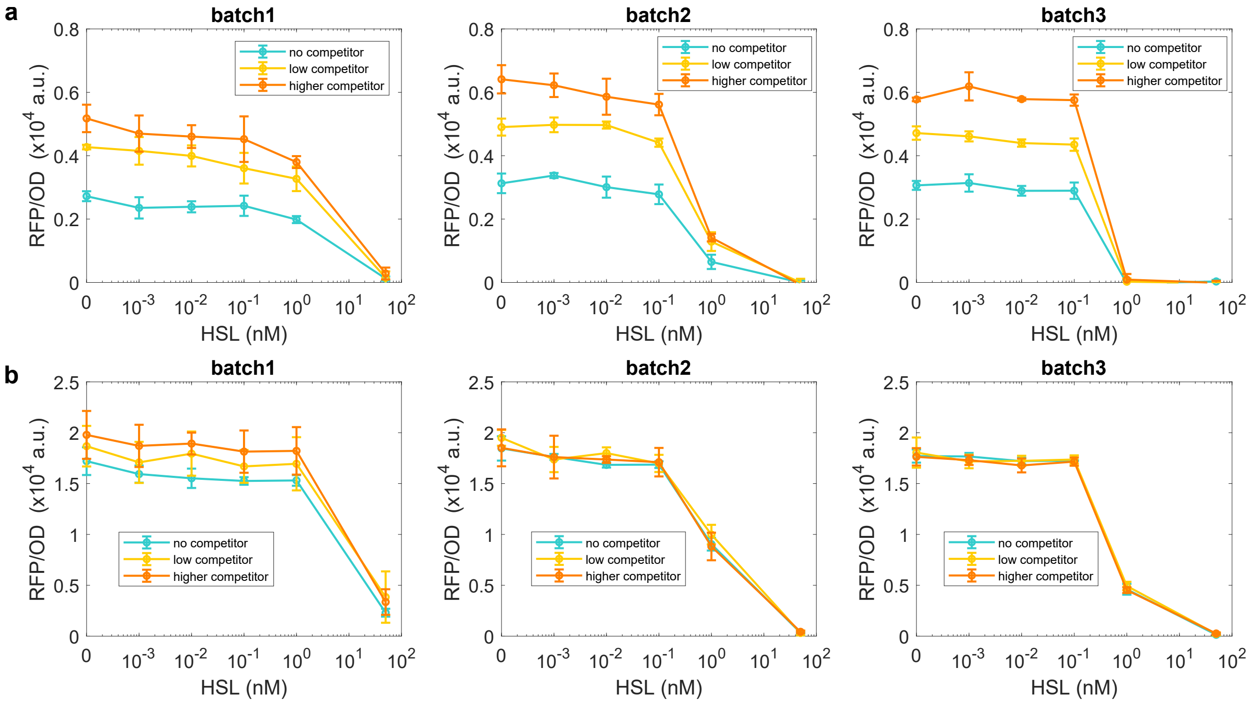

Supplementary Figure 20: **RFP/OD data for low copy unregulated (UR - panel a) and regulated (R - panel b) NOT gate in Figure 2d and 2e.** Data are presented as mean values ± SD of n=3 biologically independent experiments with microplate photometer.

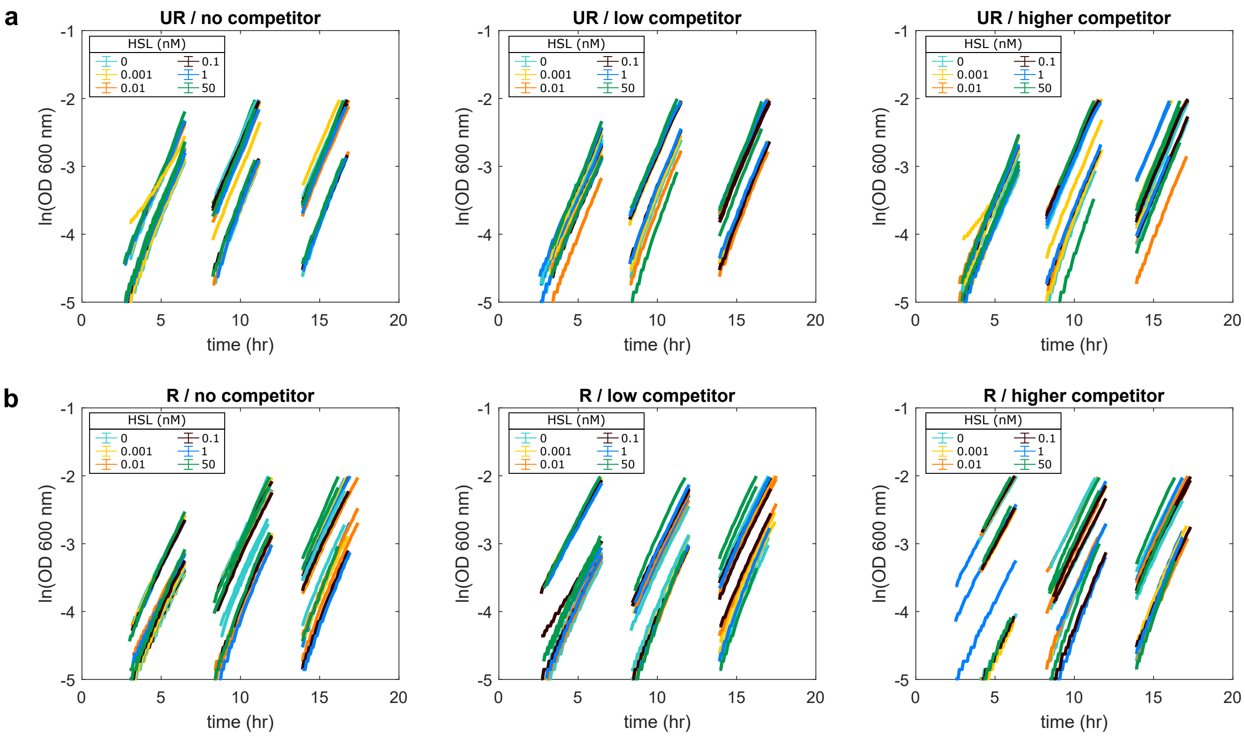

Supplementary Figure 21: **Exponential growth data for low copy unregulated (UR - panel a) and regulated (R - panel b) NOT gate in Figure 2d and 2e.** Each trace represents the growth curve of an independent experiment. Three biological replicates are shown in the same color as depicted in the figure legends. Linearity of each growth curve confirms the exponential growth. To analyze RFP/OD values, we choose the data point with an OD value most close to 0.0598 for each experimental condition.

Multiple batch data of the Cascades in the main text

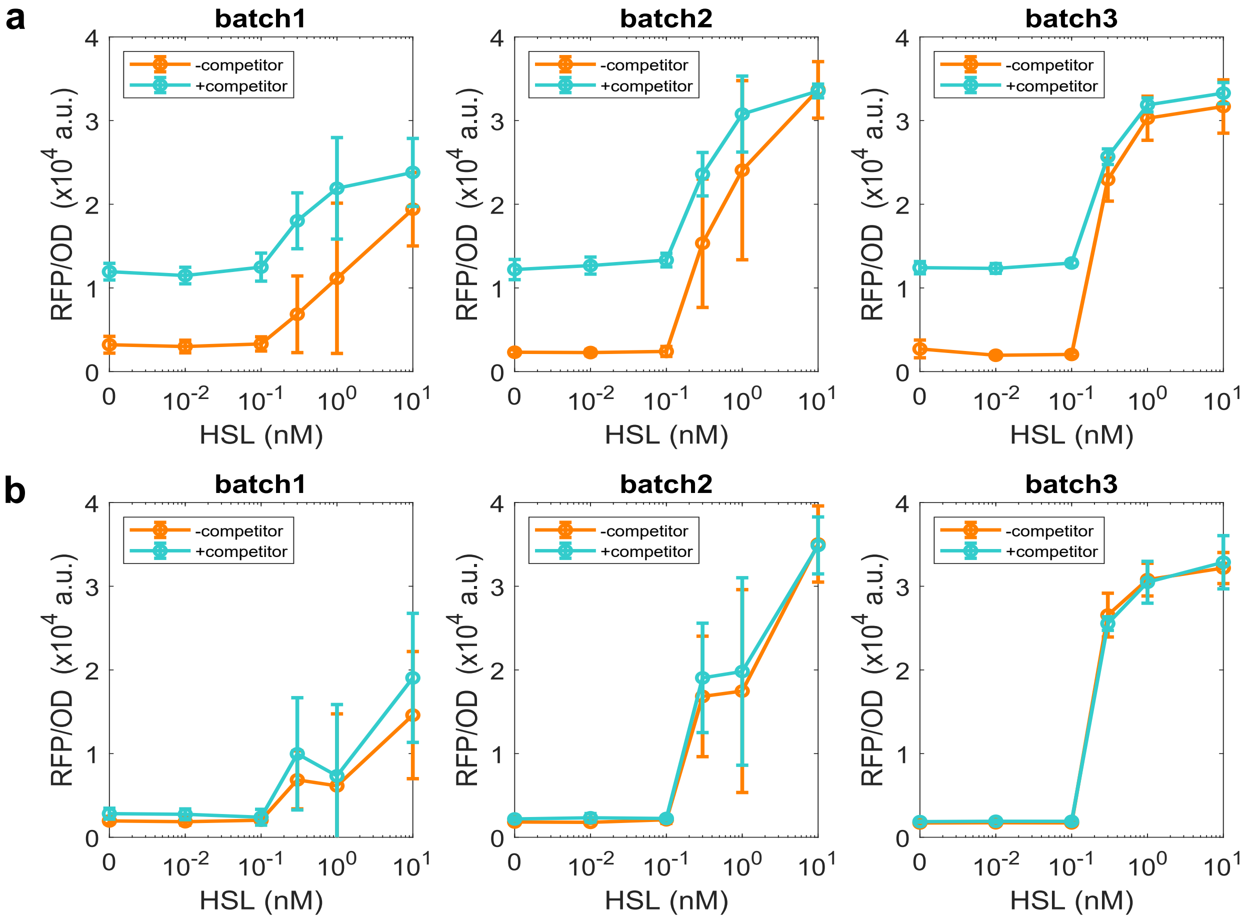

Supplementary Figure 22: **RFP/OD data for unregulated (UR - panel a) and regulated (R - panel b) Cascade in Figure 3c and 3d.** Data are presented as mean values ± SD of n=3 biologically independent experiments with microplate photometer.

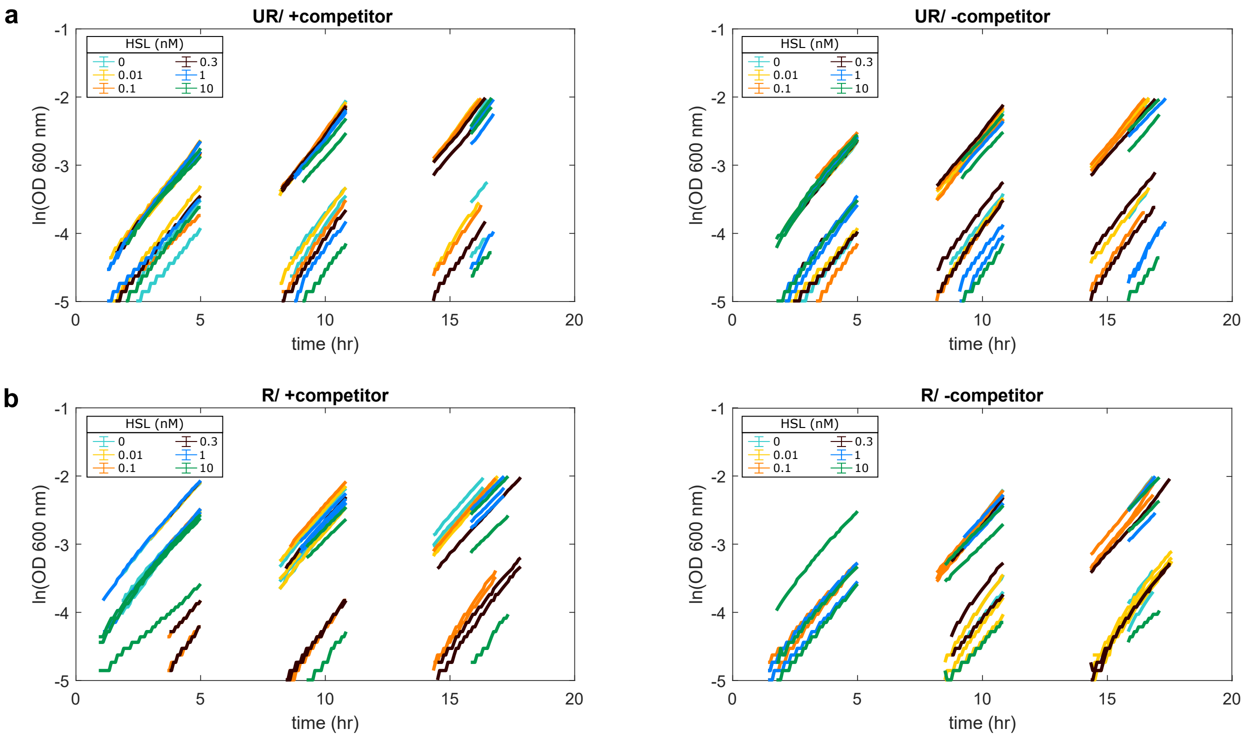

Supplementary Figure 23: **Exponential growth data for unregulated (UR - panel a) and regulated (R - panel b) Cascade in Figure 3c and 3d.** Each trace represents the growth curve of an independent experiment. Three biological replicates are shown in the same color as depicted in the figure legends. Linearity of each growth curve confirms the exponential growth. To analyze RFP/OD values, we choose the data point with an OD value most close to 0.0598 for each experimental condition.

References

[S1] Del Vecchio, D., and Murray, R. *Biomolecular feedback systems.* Princeton, 2014.

[S2] Rudin, W. *Principles of Mathematical Analysis.* MacGraw-Hill, 1976.

[S3] Mekler V., *et al.*. Kinetics of the CRISPR-Cas9 effector complex assembly and the role of 3-terminal segment of guide RNA. *Nucleic acids research* **44**,2837-2845 (2016).

[S4] Phillips, R., and Milo, R. *Cell Biology by the Numbers* Garland Science, 2015.

[S5] Bernstein, J.A., Khodursky, A.B., Lin, P.H., Lin-Chao & Cohen, S.N. Global analysis of mRNA decay and abundance in *Escherichia coli* at single-gene resolution using two-color fluorescent DNA microarrays. *Proc. Nat. Acad. Sci.* **15**, 9697-9702, 2002.

[S6] Shetty, R.P., Endy, D. & Knight, T.F. Engineering BioBrick vectors from BioBrick parts. *J. Biol. Eng.* **5**,2008.

[S7] Wright, A. V., et al. Rational design of a split-Cas9 enzyme complex. *Proc. Nat. Acad. Sci.* **112**, 2984-2989, 2015.

[S8] Sternberg, S. H., Redding, S., Jinek, M., Greene, E. C. & Doudna, J. A. DNA interrogation by the CRISPR RNA-guided endonuclease Cas9. *Nature.* **507**, 2014.

[S9] Zhang, S. & Voigt, C.A. Engineered dCas9 with reduced toxicity in bacteria: implications for genetic circuit design. *Nuc. Ac. Res.* **46**,11115-11125 (2018).

[S10] Datsenko, K.A. & Wanner, B.L. One-step inactivation of chromosomal genes in *Escherichia coli* K-12 using PCR products. *Proc. of the Nat. Ac. of Sci.* **97**,6640-6645,2000

[S11] Salis H.M., Mirsky, E.A. & Voigt C.A. Automated design of synthetic ribosome binding sites to control protein expression. *Nat. Biotech.***27**,946-950,2009

[S12] Thompson, M.G, et al. Isolation and characterization of novel mutations in the pSC101 origin that increase copy number. *Sci. Rep.***8**,(2018)

[S13] Peterson, J. & Phillips, G.J. New pSC101-derivative cloning vectors with elevated copy numbers. *Plasmid* **59**,193-201,2008

[S14] Kosuri, S., et al. Composability of regulatory sequences controlling transcription and translation in *Escherichia coli. Proc. of the Nat. Ac. of Sci.***110**,14024-14029 (2013)

[S15] Huang, H, Qian, Y. & Del Vecchio, D. A quasi-integral controller for adaptation of genetic modules to variable ribosome demand. *Nat. Comm.* **9**,2018

[S16] Yanisch-Perron, C. Vieira, J. Messing, J. Improved M13 phage cloning vectors and host strains: nucleotide sequences of the M13mp18 and pUC19 vectors. *Gene* **33**,103-119 (1985)

[S17] Bellato M., *et al.* CRISPR interference as low burden logic inverter in synthetic genetic circuits: characterization and tuning *bioRχiv* url:https://www.biorxiv.org/content/10.1101/2020.08.03.234096v1 (2020).

[S18] Reis, A.C., et al. Simultaneous repression of multiple bacterial genes using non repetitive extra-long sgRNA arrays. *Nat. Biotech.***37**,1294-1301,2019

[S19] Chen Y., et al. Characterization of 582 natural and synthetic terminators and quantification of their design constraints. *Nat. Met.***10**,659-664,2013
