# Supplementary material for: dCas9 regulator to neutralize competition in CRISPRi circuits
Source: Nat Commun. 2021 Mar 16;12:1692. doi: 10.1038/s41467-021-21772-6 (PMC7966764; doi:10.1038/s41467-021-21772-6)
Supplement: Supplementary file 4 — Reporting Summary [file 41467_2021_21772_MOESM4_ESM.pdf]

nature research

Corresponding author(s): Domitilla Del Vecchio

Last updated by author(s): Jan 27, 2021

Reporting Summary

Nature Research wishes to improve the reproducibility of the work that we publish. This form provides structure for consistency and transparency in reporting. For further information on Nature Research policies, see our Editorial Policies and the Editorial Policy Checklist.

Statistics

For all statistical analyses, confirm that the following items are present in the figure legend, table legend, main text, or Methods section.

n/a	Confirmed	
☐	☒	The exact sample size (n) for each experimental group/condition, given as a discrete number and unit of measurement
☐	☒	A statement on whether measurements were taken from distinct samples or whether the same sample was measured repeatedly
☒	☐	The statistical test(s) used AND whether they are one- or two-sided<br>*Only common tests should be described solely by name; describe more complex techniques in the Methods section.*
☒	☐	A description of all covariates tested
☒	☐	A description of any assumptions or corrections, such as tests of normality and adjustment for multiple comparisons
☐	☒	A full description of the statistical parameters including central tendency (e.g. means) or other basic estimates (e.g. regression coefficient) AND variation (e.g. standard deviation) or associated estimates of uncertainty (e.g. confidence intervals)
☒	☐	For null hypothesis testing, the test statistic (e.g. F, t, r) with confidence intervals, effect sizes, degrees of freedom and P value noted<br>*Give P values as exact values whenever suitable.*
☒	☐	For Bayesian analysis, information on the choice of priors and Markov chain Monte Carlo settings
☒	☐	For hierarchical and complex designs, identification of the appropriate level for tests and full reporting of outcomes
☒	☐	Estimates of effect sizes (e.g. Cohen's d, Pearson's r), indicating how they were calculated

Our web collection on statistics for biologists contains articles on many of the points above.

Software and code

Policy information about availability of computer code

Data collection	Gen5 v1.11.5 software was used to collect data from the microplate photometer. CFlowPlus v 1.0.264.14 software was used to collect cytometric data from the BD Accuri C6 cytometer.
Data analysis	MATLAB R2020a was used to compute mean and standard deviation for microplate photometer data. FlowJo v10 was used to analyze cytometric data. RBS calculator is the verson 2.0. The CRISPR Guide RNA Design Tool is the latest on-line version provided by Benchling.

For manuscripts utilizing custom algorithms or software that are central to the research but not yet described in published literature, software must be made available to editors and reviewers. We strongly encourage code deposition in a community repository (e.g. GitHub). See the Nature Research guidelines for submitting code & software for further information.

Data

Policy information about availability of data

All manuscripts must include a data availability statement. This statement should provide the following information, where applicable:

- Accession codes, unique identifiers, or web links for publicly available datasets
- A list of figures that have associated raw data
- A description of any restrictions on data availability

Simulation and fluorescence data generated or analyzed during this study are included in the paper and its Supplementary Information files. A reporting summary for this Article is available as a Supplementary Information file. DNA sequences of plasmids and the list of all primers are provided in Supplementary Data. The codes to produce simulation results in Figure 2 and 3 are provided in Supplementary Software. The source data underlying Figures 2-3 are provided in Source Data. We deposited pdCas9_CL and pAUX_OL plasmids to Addgene (ID 166245 and 166246, respectively).

Field-specific reporting

Please select the one below that is the best fit for your research. If you are not sure, read the appropriate sections before making your selection.

- [x] Life sciences
- [ ] Behavioural & social sciences
- [ ] Ecological, evolutionary & environmental sciences

For a reference copy of the document with all sections, see nature.com/documents/nr-reporting-summary-flat.pdf

Life sciences study design

All studies must disclose on these points even when the disclosure is negative.

Sample size	A sample size of at least n = 3 (three experimental repeats) was chosen because it is standard practice in the field. Microplate photometer measured the whole population of E coli cells cultivated in a 96-well plate. Flow cytometry measured at least 70,000 singlet events.
Data exclusions	No data were excluded from the microplate photometer data. The flow cytometric data only collected singlet events (i.e. single cells).
Replication	All attempts at replication were successful. We performed experiments independently at least two days apart.
Randomization	We randomly took cells from a population of bacterial cells grew in the same pre-culture to proceed as the control (i.e. without AHL induction) and experimental group (i.e. with AHL induction).
Blinding	N/A: Success metrics for our controller design vs the unregulated system were pre-defined (fold-changes).

Reporting for specific materials, systems and methods

We require information from authors about some types of materials, experimental systems and methods used in many studies. Here, indicate whether each material, system or method listed is relevant to your study. If you are not sure if a list item applies to your research, read the appropriate section before selecting a response.

Materials & experimental systems

n/a	Involved in the study	
x		Antibodies
x		Eukaryotic cell lines
x		Palaeontology and archaeology
x		Animals and other organisms
x		Human research participants
x		Clinical data
x		Dual use research of concern

Methods

n/a	Involved in the study	
x		ChIP-seq
	x	Flow cytometry
x		MRI-based neuroimaging

Flow Cytometry

Plots

Confirm that:

- [x] The axis labels state the marker and fluorochrome used (e.g. CD4-FITC).
- [x] The axis scales are clearly visible. Include numbers along axes only for bottom left plot of group (a 'group' is an analysis of identical markers).
- [x] All plots are contour plots with outliers or pseudocolor plots.
- [x] A numerical value for number of cells or percentage (with statistics) is provided.

Methodology

Sample preparation	E coli cells transformed with the regulated and unregulated dCas9 generators were collected from a culture grew in the microplate photometer under the indicated experimental condition. A 5-uL cell culture were taken and diluted with 200-uL 0.22um-membrane filtrated water as the sample for flow cytometry measurement.
Instrument	BD Accuri C6 Special Order 2B2LYG RUO System, 656035
Software	CFlowPlus v 1.0.264.14 Build 20110520.264.14
Cell population abundance	At least 70,000 singlet events were collected and analyzed.

Gating strategy	We only applied the preliminary singlet-event gate (i.e. FSC-H vs FSC-A) on the starting cell population. The boundary of positive events is in the diagonal direction of the FSC-H vs FSC-A scatter plot. No further gating was applied to the collected data.

☒ Tick this box to confirm that a figure exemplifying the gating strategy is provided in the Supplementary Information.
