# Supplementary material for: dCas9 regulator to neutralize competition in CRISPRi circuits
Source: Nat Commun. 2021 Mar 16;12:1692. doi: 10.1038/s41467-021-21772-6 (PMC7966764; doi:10.1038/s41467-021-21772-6)
Supplement: Supplementary file 6 — Description of Additional Supplementary Files [file 41467_2021_21772_MOESM6_ESM.pdf]

Title: Supplementary Software

Description: The zip file including codes to produce simulation results in Figures 2 and 3.

Title: Supplementary Data 1:
Description: The zip file including DNA sequences ofplasmids and the list of all primers used
